# Anti-*Helicobacter pylori* Activity and Gastroprotective Effects of Miao Sour Soup-Derived *Lactiplantibacillus plantarum* SHY0008

**DOI:** 10.3390/microorganisms14102387

**Published:** 2026-10-19

**Authors:** Zhaoxu Ren, Shiqi Xiao, Zixin Zhang, Feiyang Wang, Qingfang Kong, Yanhui Li, Chunxu Xue, Dong Liu, Haiyan Sun

**Affiliations:** School of Food and Drug, Shenzhen Polytechnic University, Shenzhen 518000, China; renzhaoxu09@163.com (Z.R.); xiaoaaaaxiao@szpu.edu.cn (S.X.); zhangzixin@szpu.edu.cn (Z.Z.); w1355461371@163.com (F.W.); kongqingfang05@163.com (Q.K.); liyanhui1@szpu.edu.cn (Y.L.); xuechunxu@outlook.com (C.X.); liudongsz@szpu.edu.cn (D.L.)

**Keywords:** *Helicobacter pylori*, *Lactiplantibacillus plantarum* SHY0008, Miao sour soup, gastric protection

## Abstract

*Helicobacter pylori* (*H. pylori*) infection is a major global gastrointestinal health concern, and increasing antibiotic resistance has prompted interest in complementary probiotic strategies. In this study, *Lactiplantibacillus plantarum* SHY0008 isolated from Miao sour soup was evaluated using integrated in vitro, AGS cell, zebrafish, murine, and genomic approaches. SHY0008 showed favorable gastrointestinal tolerance, with an acid survival rate of 39.61 ± 1.87% after 4 h under simulated gastric conditions and a relative viable recovery of 64.20 ± 6.48% after 4 h of exposure to 0.3% oxgall. SHY0008 also produced inhibition zones of 17.56 ± 0.58 mm and 17.21 ± 0.29 mm for viable and heat-treated/sonicated bacterial preparations, respectively, and reduced the *H. pylori* urease-associated phenol-red response by 54.23 ± 3.98%. In AGS cells, SHY0008 showed an adhesion rate of 17.53 ± 1.01% and reduced the urease-associated *H. pylori* adhesion signal by 48.27 ± 2.52%. In a prophylactic zebrafish model, SHY0008 pretreatment was associated with reduced intestinal *H. pylori*-associated fluorescence and histopathological injury following subsequent exposure. In mice, SHY0008 reduced gastric *H. pylori*-associated antigen and urease signals, lowered serum CXCL1/KC, and improved gastric histopathology. Whole-genome analysis identified plantaricin-associated genes, while preliminary genome-based safety screening detected no known acquired antimicrobial resistance genes or virulence-associated determinants. Collectively, these findings support SHY0008 as a promising food-derived probiotic candidate with complementary anti-*H. pylori* and gastroprotective activities.

## 1. Introduction

*Helicobacter pylori* (*H. pylori*) is a Gram-negative, microaerophilic pathogen that was first isolated from the gastric mucosa of patients with chronic gastritis by Marshall and Warren in 1982 [1]. It is one of the most prevalent chronic bacterial infections worldwide, affecting more than half of the global population, with a particularly high prevalence in developing countries [2]. Persistent *H. pylori* infection is strongly associated with chronic gastritis, peptic ulcer disease, gastric mucosal atrophy, intestinal metaplasia, and gastric cancer, and is recognized as the most important infectious risk factor for gastric carcinogenesis. Accordingly, the World Health Organization has classified *H. pylori* as a Group I carcinogen [3].

Successful colonization and persistence of *H. pylori* in the hostile gastric environment depend on multiple virulence-associated mechanisms. Urease hydrolyzes urea into ammonia, thereby locally neutralizing gastric acidity and facilitating bacterial survival [4]. Adhesins promote attachment to gastric epithelial cells and contribute to persistent colonization [5,6]. In addition, the cytotoxin-associated gene A (CagA) and vacuolating cytotoxin A (VacA), two major *H. pylori* virulence factors, contribute to epithelial injury and chronic inflammation by activating host signaling pathways, including nuclear factor-κB (NF-κB), and promoting the production of pro-inflammatory mediators such as IL-8 [7,8,9]. These pathogenic mechanisms make bacterial colonization, adhesion, virulence-associated activity, and host inflammatory responses important targets for the development of novel strategies against *H. pylori* infection.

Probiotics have attracted considerable attention as potential adjunctive strategies for the management of *H. pylori* infection. Their anti-*H. pylori* effects are strain-dependent and may involve multiple mechanisms, including the production of organic acids and bacteriocins, direct inhibition of bacterial growth, competition for epithelial adhesion sites, interference with bacterial colonization, modulation of host inflammatory responses, and maintenance of gastrointestinal barrier function. Clinical studies have further suggested that probiotic supplementation may improve *H. pylori* eradication outcomes and reduce treatment-associated gastrointestinal adverse effects. Accordingly, probiotics have been considered as potential adjuncts to standard eradication therapy [10]. Several individual probiotic strains have also demonstrated anti-*H. pylori* activity. Chen et al. [11], for example, reported that *Lacticaseibacillus rhamnosus* GMNL-74 and *Lactobacillus acidophilus* GMNL-185 significantly reduced the adhesion of *H. pylori* to AGS gastric epithelial cells. Urrutia-Baca et al. [12] demonstrated that reuterin obtained from *Limosilactobacillus reuteri* fermentation products exhibited antibacterial activity against multiple *H. pylori* strains. Yu et al. [13] further reported that *Lacticaseibacillus casei* T1 alleviated gastric inflammation in *H. pylori*-infected mice, partly through modulation of the NF-κB signaling pathway. These findings indicate that probiotics may act against *H. pylori* through complementary antimicrobial and host-modulatory mechanisms. Nevertheless, their efficacy is highly strain-specific, emphasizing the importance of identifying and characterizing new probiotic candidates with potent anti-*H. pylori* activity.

Traditional fermented foods are important reservoirs of diverse microorganisms and may provide valuable sources of novel probiotic strains. Miao sour soup, a representative naturally fermented food from Guizhou Province, China, harbors a complex microbial community in which lactic acid bacteria are abundant. Previous studies have shown that microorganisms isolated from Miao sour soup may exhibit various beneficial properties, including antioxidant, anti-inflammatory, and gut microbiota-modulating activities [14]. However, the anti-*H. pylori* potential of microorganisms derived from Miao sour soup remains poorly characterized. In particular, whether strains isolated from this traditional fermented food can exert anti-*H. pylori* effects across different biological levels and protect against *H. pylori*-associated gastric injury remains unclear. Therefore, the present study isolated and identified a potential anti-*H. pylori* probiotic strain, *Lactiplantibacillus plantarum* SHY0008, from Miao sour soup. The probiotic characteristics of SHY0008, including tolerance to simulated gastrointestinal conditions, auto-aggregation, and in vitro antagonistic activity against *H. pylori*, were first evaluated. Its anti-*H. pylori* activity was subsequently investigated using complementary AGS cell, zebrafish, and murine infection models. The effects of SHY0008 on *H. pylori*-associated bacterial signals and host inflammatory and tissue responses were further assessed. This study provides evidence supporting Miao sour soup as a promising source of probiotic microorganisms and identifies SHY0008 as a potential probiotic candidate for mitigating *H. pylori*-associated gastric injury.

## 2. Materials and Methods

### 2.1. Bacterial Strains

*Lactiplantibacillus plantarum* SHY0008 was isolated from a single batch of naturally fermented Miao sour soup collected in Guizhou Province, China. The isolate was deposited in the Guangdong Microbial Culture Collection Center (GDMCC, Guangzhou, China) under the accession number GDMCC No. 65911. *Streptococcus thermophilus* FP13 was used as a reference probiotic comparator throughout this study. FP13 was purchased from Weikai Haisi (Shandong) Biotechnology Co., Ltd. (Zibo, China), and its previously reported anti-*H. pylori* activity is described in Chinese Patent CN116103197B [15]. Because peer-reviewed evidence specifically evaluating FP13 against *H. pylori* is limited, FP13 was not considered a validated pharmacological positive control. Nevertheless, peer-reviewed studies have reported anti-*H. pylori*-related effects for other *S. thermophilus* strains. In particular, *S. thermophilus* CRL1190 has been shown to reduce *H. pylori* adhesion and attenuate *H. pylori*-associated inflammatory responses in gastric epithelial models, supporting the biological relevance of *S. thermophilus* as a probiotic comparator in *H. pylori*-related studies [16]. *H. pylori* ATCC 43504 was purchased from Shanghai Beinuo Biotechnology Ltd. (Shanghai, China) and was used for all antibacterial, cell-based, and animal experiments.

### 2.2. Culture Media

Commercial de Man–Rogosa–Sharpe (MRS) broth and MRS agar (Guangdong Huankai Microbial Sci. & Tech. Co., Ltd., Guangzhou, China) were used for the isolation and routine cultivation of lactic acid bacteria. The compositions of the liquid and solid MRS media are listed in Tables S1 and S2, respectively. *H. pylori* was cultured on Columbia Blood Agar plates and in *H. pylori* Broth (Qingdao Hi-tech Industrial Park Hope Bio-technology Co., Ltd., Qingdao, China). Detailed formulations and preparation procedures for the solid and liquid media are provided in Tables S3 and S4, respectively.

### 2.3. Cell Line and Culture

The human gastric adenocarcinoma cell line AGS was purchased from Servicebio Technology Co., Ltd. (Wuhan, China). AGS cells were maintained in complete culture medium, while the experimental culture medium used for subsequent cell-based assays was prepared according to the formulations listed in Table S5.

### 2.4. Experimental Animals

Wild-type zebrafish were provided by the Aquatic Animal Center of Hangzhou Hunter Biotechnology Co., Ltd. (Hangzhou, China). Zebrafish were maintained in system water at 28 ± 0.5 °C under standard laboratory conditions. The culture water was prepared by dissolving 200 mg of instant sea salt in 1 L of reverse osmosis water, with a conductivity of 450–550 μS/cm, a pH of 6.5–8.5, and a hardness of 50–100 mg/L CaCO_3_. The zebrafish facility was licensed for laboratory animal use (License No. SYXK (Zhe) 2022-0004) and operated in accordance with the standards of the Association for Assessment and Accreditation of Laboratory Animal Care International (AAALAC International; Accreditation No. 001458). All experimental procedures involving zebrafish were approved by the Institutional Animal Care and Use Committee (IACUC) under protocol number IACUC-2026-202601070016-01. Six-week-old female BALB/c mice were purchased from Guangzhou Institute of Microbiology Group Co., Ltd. (Guangzhou, China). All animal experiments were reviewed and approved by the Laboratory Animal Ethics Committee of Guangdong Huawei Testing Co., Ltd., with the ethical approval number 20260403. All animal operations strictly complied with the 3R principles for laboratory animal welfare. Mice were housed under specific pathogen-free (SPF) conditions with free access to standard chow and sterile water prior to experimentation.

### 2.5. Isolation of Lactic Acid Bacteria (LAB) from Miao Sour Soup

Frozen Miao sour soup samples were thawed to room temperature and thoroughly homogenized before use. Briefly, 500 μL of the original sample was transferred into 4.5 mL of sterile phosphate-buffered saline (PBS) to obtain a 10^−1^ dilution. Ten-fold serial dilutions were subsequently prepared, and aliquots (100 μL) of the 10^−4^, 10^−5^, and 10^−6^ dilutions were spread onto MRS agar plates in triplicate. The plates were incubated at 37 °C for 48 h. Colonies exhibiting distinct morphological characteristics were selected and repeatedly streaked on fresh MRS agar plates until pure colonies with uniform morphology were obtained. The isolates were preliminarily identified by Gram staining and the catalase test. Gram-positive and catalase-negative isolates were inoculated into 5 mL of MRS broth and cultured at 37 °C with shaking at 150 rpm for 16 h to prepare seed cultures. The resulting cultures were mixed with an equal volume of sterile 50% (*v*/*v*) glycerol and stored at −80 °C for long-term preservation. A total of 12 Gram-positive and catalase-negative LAB isolates were recovered and designated SHY0001–SHY0012. These isolates were preserved for subsequent molecular identification and preliminary functional screening. Their phenotypic and taxonomic characteristics are summarized in Table S6.

### 2.6. Molecular Biological Identification

Genomic DNA was extracted from each of the 12 isolates using a bacterial genomic DNA extraction kit according to the manufacturer’s instructions. The nearly full-length 16S rRNA gene was amplified using the universal bacterial primers 27F and 1492R. PCR amplification was carried out in a final reaction volume of 40 μL, containing 20 μL PCR Mix, 0.8 μL each of the forward and reverse primers, 2 μL of genomic DNA template, and 16.4 μL of nuclease-free water. The PCR program consisted of an initial denaturation at 96 °C for 5 min, followed by 35 cycles of denaturation at 96 °C for 30 s, annealing at 56 °C for 30 s, and extension at 72 °C for 1 min, with a final extension at 72 °C for 5 min. The PCR products were analyzed by 1.0% agarose gel electrophoresis to confirm the expected amplicon size. The amplified products were subsequently sequenced by Sangon Biotech Co., Ltd. (Shanghai, China). The obtained sequences were compared with those available in the National Center for Biotechnology Information (NCBI) database using the Basic Local Alignment Search Tool (BLAST https://blast.ncbi.nlm.nih.gov/Blast.cgi, accessed on 30 July 2026) for taxonomic identification.

### 2.7. Preliminary Screening Based on H. pylori Urease Inhibition

All 12 LAB isolates were subjected to preliminary screening based on their ability to inhibit *H. pylori* urease activity. Urease inhibition was used solely as a primary screening criterion for selecting a candidate strain for subsequent comprehensive evaluation. For each isolate, six technical replicate wells were analyzed within a single screening experiment. The *H. pylori* control was included in parallel, and the urease inhibition rate was calculated relative to the corresponding *H. pylori* control. The preliminary screening data were used for candidate selection and were not treated as independent biological replicates for inferential statistical comparison. SHY0008 showed the highest mean urease inhibitory activity among the 12 isolates and was therefore selected for subsequent characterization. Preliminary screening of LAB isolates based on inhibition of *H. pylori* urease activity is provided in Table S7.

### 2.8. Preparation of Bacterial Suspensions and Cell Samples

Preparation of Probiotic Suspensions. Frozen stocks of FP13 and SHY0008 preserved at −80 °C were revived by inoculating 2% (*v*/*v*) of each culture into 5 mL of MRS broth and incubating at 37 °C for 16 h. The strains were subcultured three consecutive times prior to use. For probiotic suspension preparation, activated seed cultures at the exponential growth phase were inoculated into fresh MRS broth at 2% (*v*/*v*) and incubated at 37 °C for 16 h. The cultures were centrifuged at 5000 rpm for 10 min at 4 °C, and the supernatants were discarded. The bacterial pellets were washed twice with sterile PBS and resuspended in sterile PBS. Viable cell concentrations were determined by plate counting and adjusted with sterile PBS to the concentration required for each subsequent assay.

Preparation of Heat-Treated and Sonicated Bacterial Preparations. Probiotic cultures were incubated in a 65 °C water bath for 30 min and subsequently subjected to ultrasonic disruption at 50% output power for a total of 30 min using 2 s pulse-on and 3 s pulse-off cycles. The resulting preparations, which contained heat-treated and sonication-disrupted bacterial material, were designated as heat-treated and sonicated bacterial preparations (HTSBPs).

Preparation of *H. pylori* Suspension. Frozen glycerol stocks of *H. pylori* stored at −80 °C were thawed in a 37 °C water bath and inoculated into *H. pylori* broth at 2% (*v*/*v*). Cultures were incubated under microaerophilic conditions (5% O_2_, 10% CO_2_, and 85% N_2_) at 37 °C with shaking for 48–72 h. The strain was subcultured three consecutive times to ensure complete recovery. The bacterial cells were harvested by centrifugation, washed twice with sterile PBS, and resuspended in sterile PBS. The bacterial concentration was determined by viable plate counting, and the suspension was adjusted to the desired concentration before subsequent experiments.

Cell Culture. Cryopreserved AGS cells were removed from liquid nitrogen and rapidly thawed using a programmed cell thawing device. After the exterior of the cryovial was disinfected with 75% ethanol, the cell suspension was transferred into a sterile 15-mL centrifuge tube containing 3 mL of complete AGS culture medium. Cells were centrifuged at 1500 rpm for 5 min at room temperature, and the supernatant was discarded. The cell pellet was resuspended in 3 mL of complete culture medium by gentle pipetting and transferred into a T25 cell culture flask. Fresh complete culture medium was added to the desired volume, and the cells were maintained in a humidified incubator at 37 °C with 5% CO_2_ until reaching the required confluence for subsequent experiments.

### 2.9. Acid Tolerance Assay

The acid tolerance of the probiotic strains was evaluated according to the method described by Haghshenas et al. [17] with minor modifications. The acid tolerance of FP13 and SHY0008 was evaluated under simulated gastric conditions. The artificial gastric juice was adjusted to pH 2.0 before use. Equal volumes of bacterial suspension and artificial gastric juice were mixed, and the pH of the mixed exposure system was measured immediately after mixing. The final pH values were 2.39 ± 0.03 for FP13 and 2.39 ± 0.02 for SHY0008. The mixtures were then incubated at 37 °C for 4 h. Following treatment, the bacterial suspensions were serially diluted with PBS, and appropriate dilutions were spread onto MRS agar plates for viable counting.

The assay was independently performed three times for each strain using independently prepared bacterial cultures (n = 3 biological replicates per strain). For each biological replicate, viable counts were determined using three technical replicate agar plates. Colony counts from the three plates were averaged to obtain a single CFU/mL value for each biological replicate. Survival rates were calculated independently for each biological replicate, and only the three biological replicate values were used for statistical analysis.

The composition of the artificial gastric juice is provided in File S1. The survival rate of each strain after artificial gastric juice treatment was calculated using the following equation:

$$Survival\;rate\;(\%)\;=\frac{N_{t}}{N_{0}}\times 100\%$$

where N_0_ is the initial viable cell concentration (CFU/mL) before treatment, and N_t_ is the viable cell concentration (CFU/mL) after treatment, determined by the plate count method.

### 2.10. Bile Salt Tolerance Assay

The bile salt tolerance of the probiotic strains was evaluated according to the method described by Zhou et al. [18] with minor modifications. The bile salt tolerance of FP13 and SHY0008 was evaluated using MRS broth supplemented with 0.3% (*w*/*v*) oxgall bile salts. Briefly, bacterial suspensions were inoculated into bile salt-containing MRS broth and incubated at 37 °C for 4 h. Following incubation, viable bacterial counts were determined by the plate-count method. The assay was independently performed three times for each strain using independently prepared bacterial cultures (n = 3 biological replicates per strain). For each biological replicate, viable counts were determined using three technical replicate agar plates. Colony counts from the three plates were averaged to obtain a single CFU/mL value for each biological replicate. Survival rates were calculated independently for each biological replicate, and only the three biological replicate values were used for statistical analysis. The percentage of viable cells recovered after bile exposure was calculated relative to the viable count immediately before treatment as follows:

$$\mathrm{Relative}\;\mathrm{viable}\;\mathrm{recovery}\;(\%)\;=\frac{N_{t}}{N_{0}}\times 100\%$$

where N_0_ represents the viable cell concentration immediately before exposure to 0.3% oxgall, and N_t_ represents the viable cell concentration after 4 h of exposure. A bile-free growth control was not used in this calculation; therefore, this endpoint represents relative viable recovery following short-term bile exposure rather than a growth ratio relative to bile-free MRS.

### 2.11. Auto-Aggregation Assay

The auto-aggregation ability of the probiotic strains was determined by measuring the decrease in the optical density of the upper suspension during static incubation. Briefly, bacterial suspensions of FP13 and SHY0008 were incubated statically at 37 °C for 12 h. At 0, 4, 8, and 12 h, the absorbance of the upper suspension was measured at 600 nm (OD_600_) using a microplate reader. For each strain, three technical replicate wells were prepared from the same bacterial suspension and monitored at 0, 4, 8, and 12 h. Auto-aggregation rates were calculated for each technical replicate well. The area under the OD_600_-time curve (AUC) was also calculated separately for each technical replicate. Because all three wells were derived from the same bacterial preparation, they were considered technical replicates rather than independent biological replicates. Accordingly, the auto-aggregation data were summarized descriptively and were not subjected to inferential statistical analysis. The auto-aggregation rate was calculated using the following equation:

$$Auto\text{-}aggregation\;rate\;\left(\%\right)=\frac{A_{0}-A_{t}}{A_{0}}\;\times \;100\%$$

where A_0_ represents the absorbance of the upper suspension at 0 h, and A_t_ represents the absorbance of the upper suspension after incubation for 4, 8, or 12 h.

### 2.12. Mixed-Suspension Aggregation Assay with H. pylori

The aggregation behavior of mixed suspensions containing FP13 or SHY0008 and *H. pylori* was evaluated by monitoring the decrease in OD_600_ during static incubation. Briefly, bacterial suspensions of FP13, SHY0008, and *H. pylori* were adjusted to a final concentration of 1 × 10^8^ CFU/mL. Equal volumes of the probiotic suspension and *H. pylori* suspension were mixed thoroughly by vortexing for 1 min and then incubated statically at 37 °C for 12 h. At 0, 2, 4, 8, and 12 h, the absorbance of the upper suspension was measured at OD_600_ using a microplate reader. For each probiotic–*H. pylori* combination, three technical replicate wells were prepared from the same bacterial suspensions and monitored over time.

The percentage decrease in OD600 of the mixed suspension was calculated relative to the initial OD_600_ at 0 h. Because single-strain auto-aggregation controls for the probiotic strains and *H. pylori* were not run in parallel with the mixed-suspension assay, the resulting values do not represent corrected mixed-suspension aggregation after accounting for independent sedimentation of the individual strains. Therefore, these measurements were interpreted descriptively as mixed-suspension aggregation/sedimentation. The three wells were considered technical replicates and were not treated as independent biological replicates or subjected to inferential statistical analysis.

The mixed-suspension aggregation was calculated according to the following equation:

$$Mixed\text{-}suspension\;aggregation\;(\%)=\frac{A_{0}-A_{t}}{A_{0}}\;\times 100\%$$

A_0_ represents the OD600 of the mixed suspension at 0 h, and A_t_ represents the OD600 after 2, 4, 8, or 12 h of static incubation.

### 2.13. Cell Surface Hydrophobicity Assay

The cell surface hydrophobicity of the probiotic strains was evaluated using the microbial adhesion to hydrocarbons (MATH) method. Briefly, bacterial suspensions of FP13 and SHY0008 were adjusted to a final concentration of 1 × 10^9^ CFU/mL. Subsequently, 1 mL of xylene was added to 4 mL of each bacterial suspension (4:1, *v*/*v*). The mixtures were vortexed vigorously for 2 min and then allowed to stand at 37 °C for 15 min to facilitate phase separation. After separation, the absorbance of the lower aqueous phase was measured at OD_600_ using a microplate reader. The cell surface hydrophobicity was calculated using the following equation:

$$Hydrophobicity\;\left(\%\right)=\frac{A_{0}-A_{t}}{A_{0}}\times 100\%$$

where A_0_ represents the initial absorbance of the bacterial suspension before the addition of xylene, and A_t_ represents the absorbance of the lower aqueous phase after phase separation. The cell surface hydrophobicity assay was independently performed three times for each strain using independently prepared bacterial cultures (n = 3 biological replicates per strain). For each biological replicate, three technical replicate wells were measured before and after xylene treatment. The technical replicate OD600 values were averaged to obtain a single initial absorbance (A_0_) and post-treatment absorbance (A_t_) for each biological replicate. Hydrophobicity was then calculated independently for each biological replicate, and only the three biological replicate values were used for statistical analysis.

### 2.14. Urease Inhibitory Activity Assay

The inhibitory effect of the probiotic strains on the urease activity of *H. pylori* was evaluated using a modified phenol red assay. Briefly, the bacterial suspensions of FP13 and SHY0008 were adjusted to 1 × 10^9^ CFU/mL, whereas the *H. pylori* suspension was adjusted to 1 × 10^8^ CFU/mL using *H. pylori* broth supplemented with 5% (*v*/*v*) sterile defibrinated sheep blood. The probiotic suspension and *H. pylori* suspension were mixed at a 1:4 (*v*/*v*) ratio in sterile 1.5-mL microcentrifuge tubes. The mixtures were incubated under microaerophilic conditions at 37 °C for 24 h.

Following incubation, urease activity was determined in a 96-well microplate. Four experimental groups were included: (i) *H. pylori* positive control, (ii) FP13 + *H. pylori*, (iii) SHY0008 + *H. pylori*, and (iv) broth blank control. For the positive control, 50 μL of the *H. pylori* culture was mixed with 150 μL of urease indicator solution. For the experimental groups, 50 μL of the corresponding mixed culture was added to 150 μL of urease indicator solution. For the blank control, 50 μL of sterile *H. pylori* broth was mixed with 150 μL of urease indicator solution. The reaction mixtures were thoroughly mixed and incubated at room temperature for 15 min, after which the absorbance was measured at 561 nm using a microplate reader.

The experiment was independently performed three times using independently prepared bacterial cultures (n = 3 biological replicates). Within each independent experiment, each treatment was measured in three technical replicate wells. The three technical measurements were averaged to generate a single value for each biological replicate, and only the biological replicate values were used for statistical analysis.

The composition of the urease indicator solution is provided in Table S8. The urease inhibition rate was calculated using the following equation:

$$Urease\;inhibition\;rate\;(\%)=\frac{A_{H}-A_{M}}{A_{H}}\times 100\%$$

where A_H_ represents the absorbance of the *H. pylori* positive control after 24 h of incubation, and A_M_ represents the absorbance of the corresponding probiotic-treated group after 24 h of incubation.

### 2.15. In Vitro Anti-H. pylori Activity Assays

#### 2.15.1. Agar Well Diffusion Assay

The antibacterial activity of FP13 and SHY0008 against *H. pylori* was evaluated using the Oxford cup diffusion assay.

Briefly, FP13, SHY0008, and *H. pylori* cells were harvested from their respective culture media, washed twice with sterile PBS, and resuspended in sterile PBS. The *H. pylori* suspension was adjusted to 1 × 10^5^ CFU/mL, whereas the probiotic suspensions were adjusted to 1 × 10^9^ CFU/mL. An aliquot of 100 μL of the *H. pylori* suspension was evenly spread onto Columbia blood agar plates using a sterile disposable spreader to form a uniform bacterial lawn.

Sterile Oxford cups (8 mm in inner diameter) were carefully placed on the agar surface. Sterile PBS was included as the vehicle control to exclude any inhibitory effect attributable to the suspension medium. Subsequently, 200 μL of PBS-resuspended FP13 viable bacterial suspension (VBS), SHY0008 VBS, or the corresponding HTSBP was added to each Oxford cup. The pH values of the PBS-resuspended VBS, HTSBP, and sterile PBS control were measured immediately before use. The mean pH values were 6.62 ± 0.04 and 6.66 ± 0.06 for FP13-VBS and SHY0008-VBS, respectively, and 6.57 ± 0.04 and 6.55 ± 0.02 for FP13-HTSBP and SHY0008-HTSBP, respectively. Sterile PBS (pH 6.80) was used as the negative control. For each bacterial preparation, pH values were obtained from three independently prepared samples, each measured in triplicate. The assay was independently performed three times using independently prepared bacterial cultures (n = 3 biological replicates). In each independent experiment, one agar plate was used for each treatment, and the inhibition zone diameter obtained from each independent experiment was treated as one biological replicate for statistical analysis. The plates were incubated under microaerophilic conditions at 37 °C for 24 h. After incubation, the diameter of the inhibition zone surrounding each Oxford cup was measured in mm using a digital vernier caliper.

#### 2.15.2. Effects of Cell-Free Supernatants on *H. pylori* Culture Optical Density

Cell-free supernatants (CFS) of the probiotic strains were prepared as follows. Fully activated cultures of FP13 and SHY0008 were centrifuged at 5000 rpm for 10 min at 4 °C. The supernatants were collected and sterilized by filtration through a 0.22-μm membrane filter, which were designated as the native CFS (NCFS). The pH values of the freshly prepared NCFS samples were measured using a calibrated pH meter before pH adjustment. The measurements were performed using three independently prepared CFS samples (n = 3 biological replicates), with each sample measured in triplicate. Technical measurements were averaged within each biological replicate. The mean pH values were 4.06 ± 0.09 for FP13-NCFS and 4.02 ± 0.07 for SHY0008-NCFS. A portion of each supernatant was subsequently adjusted to pH 6.8 using sterile NaOH and designated as the pH-adjusted CFS (PACFS).

To evaluate the inhibitory activity against *H. pylori*, the NCFS or PACFS was mixed with the *H. pylori* suspension at a 1:9 (*v*/*v*) ratio and incubated under microaerophilic conditions at 37 °C for 6 h. A culture of *H. pylori* without CFS served as the positive control. Following incubation, the absorbance was measured at 600 nm using a microplate reader.

The assay was independently performed three times using independently cultured bacterial preparations and separately prepared CFS samples (n = 3 biological replicates). Within each independent experiment, each treatment was measured in three technical replicate wells. Technical replicate measurements were averaged to obtain a single OD600 value for each biological replicate, and only the biological replicate values were used to calculate growth inhibition rates and for statistical analysis. The growth inhibition rate of *H. pylori* was calculated using the following equation:

$$Growth\;inhibition\;rate\;(\%)=\frac{A_{H}-A_{F}}{A_{H}}\times 100\%$$

where A_H_ represents the absorbance of the *H. pylori* positive control after 6 h of incubation, and A_F_ represents the absorbance of the corresponding CFS-treated group after 6 h of incubation.

#### 2.15.3. Effects of CFS on *H. pylori* Membrane Integrity Assessed by LIVE/DEAD Staining

Three types of probiotic CFSs were prepared, including NCFS, PACFS, and heat-inactivated cell-free supernatant (HICFS). The HI-CFS was prepared by centrifuging the heat-inactivated probiotic culture at 5000 rpm for 10 min at 4 °C, followed by sterilization of the collected supernatant through a 0.22-μm membrane filter. The NCFS, PACFS, and HICFS were individually mixed with a *H. pylori* suspension (1 × 10^8^ CFU/mL) at a 1:9 (*v*/*v*) ratio and co-incubated under microaerophilic conditions at 37 °C for 6 h. Following incubation, bacterial viability was evaluated using a LIVE/DEAD bacterial fluorescence staining assay. The stained bacterial suspensions were analyzed using a JSY-FL-054 microbial cell counter (BodBoge, Guangzhou, China). The instrument distinguishes viable and dead bacterial cells according to their fluorescence signals, with green fluorescence representing viable cells and red fluorescence representing dead cells. The numbers of viable and dead bacterial events were automatically detected and counted by the instrument, and the dead-cell percentage was directly calculated and output by the instrument based on the relative proportions of red- and green-fluorescent bacterial events. Representative fluorescence images generated during the analysis were used for visualization only and were not used as the basis for the quantitative dead-cell percentages. The LIVE/DEAD fluorescence assay was independently performed three times using independently prepared CFS samples and independently prepared *H. pylori* cultures (n = 3 biological replicates). The instrument-derived dead-cell percentage from each independent experiment was treated as one biological replicate, and only the three independent biological replicate values were used for statistical analysis.

### 2.16. AGS Cell-Based Adhesion and Inflammation Assays

#### 2.16.1. Adhesion to AGS Cells

FP13 and SHY0008 cultures were centrifuged at 5000 rpm for 10 min at 4 °C. The supernatants were discarded, and the bacterial pellets were washed twice with sterile PBS. The bacteria were then resuspended in AGS cell culture medium and adjusted to a final concentration of 1 × 10^8^ CFU/mL. AGS cells were seeded into 6-well culture plates and cultured until a confluent monolayer was formed. The culture medium was removed, and the cell monolayers were washed twice with sterile PBS. Subsequently, 1 mL of the bacterial suspension was added to each well, and the plates were incubated at 37 °C in a humidified atmosphere containing 5% CO_2_ for 4 h. The adhesion assay was independently performed three times using independently cultured AGS cells and independently prepared bacterial suspensions (n = 3 biological replicates per strain). Within each independent experiment, adherent bacteria were quantified using three technical replicate measurements. Technical replicate values were averaged to obtain a single adhesion rate for each biological replicate, and only the three biological replicate values were used for statistical analysis. Following incubation, the bacterial suspension was removed, and the cell monolayers were washed twice with sterile PBS to remove non-adherent bacteria. Subsequently, 0.4 mL of TrypLE™ Express was added to each well, and the cells were incubated at 37 °C for 5 min. Cell digestion was terminated by adding 0.6 mL of AGS cell culture medium. The cell suspension was collected, centrifuged at 1500 rpm for 5 min at room temperature, and the pellet was resuspended in sterile PBS. Serial ten-fold dilutions were prepared, and the number of adherent bacteria was determined by the plate count method. The adhesion rate was calculated using the following equation:

$$\mathrm{Adhesion}\;\mathrm{rate}\;(\%)=\frac{N_{t}}{N_{0}}\times 100\%$$

where N_0_ is the initial bacterial concentration (CFU/mL) added to the AGS cells, and N_t_ is the concentration of adherent bacteria (CFU/mL) recovered after incubation.

#### 2.16.2. Urease-Based Assessment of *H. pylori* Adhesion to AGS Cells

FP13 and SHY0008 bacterial suspensions were resuspended in AGS cell culture medium and adjusted to a final concentration of 1 × 10^8^ CFU/mL.

AGS cells were seeded into 6-well plates and cultured until a confluent monolayer was formed. The culture medium was removed, and the cell monolayers were washed twice with sterile PBS.

The experiment consisted of two groups: an experimental group and a control group. In the experimental group, FP13 or SHY0008 and *H. pylori* suspensions were simultaneously added to the AGS cell monolayers to achieve a multiplicity of infection (MOI) of 100 for both bacterial strains. In the control group, only the *H. pylori* suspension was added to the AGS cells under identical conditions. The plates were incubated at 37 °C in a humidified atmosphere containing 5% CO_2_ for 2 h. After incubation, the culture medium was removed, and the AGS monolayers were washed twice with 1 mL of sterile PBS to remove non-adherent bacteria. Subsequently, 1.5 mL of urease indicator solution was added to each well, and the absorbance was measured at 561 nm using a microplate reader. Because *H. pylori* expresses strong urease activity, the residual urease-associated signal after removal of non-adherent bacteria was used as an indirect surrogate for cell-associated *H. pylori*. This colorimetric endpoint therefore provides a relative estimate of *H. pylori* adhesion and does not directly quantify viable adherent bacteria. The relative inhibition of the urease-associated adhesion signal was calculated as follows:

$$\mathrm{Adhesion}\;\mathrm{inhibition}\;\mathrm{rate}\;(\%)=\frac{{\mathrm{OD}}_{\mathrm{EG}}}{{\mathrm{OD}}_{\mathrm{CG}}}\times 100\%$$

where OD_EG_ represents the OD_561_ of wells co-incubated with *H. pylori* and the probiotic strain, and OD_CG_ represents the OD_561_ of wells inoculated with *H. pylori* alone. The *H. pylori* adhesion inhibition assay was independently performed three times using independently cultured AGS cells and independently prepared bacterial suspensions (n = 3 biological replicates). Within each independent experiment, each treatment was measured in three technical replicate wells. Technical replicate measurements were averaged to obtain a single OD_561_ value for each biological replicate. Adhesion inhibition rates were calculated using the corresponding control value from the same independent experiment, and only the three biological replicate values were used for statistical analysis.

#### 2.16.3. IL-8 Expression in *H. pylori*-Infected AGS Cells

The anti-inflammatory effects of FP13 and SHY0008 on *H. pylori*-induced AGS cells were evaluated by measuring the secretion of IL-8.

FP13, SHY0008, and *H. pylori* were resuspended in AGS cell culture medium and adjusted to a final concentration of 1 × 10^8^ CFU/mL. AGS cells were harvested by enzymatic digestion, seeded into 6-well plates and cultured until a confluent monolayer was established. FP13 or SHY0008 and *H. pylori* suspensions were simultaneously added to the AGS cell monolayers at a MOI of 100 for each bacterial strain. The plates were incubated at 37 °C in a humidified atmosphere containing 5% CO_2_ for 24 h. Three experimental groups were established: (i) probiotic-treated group, in which AGS cells were co-incubated with the probiotic strain and *H. pylori*; (ii) positive control group, in which AGS cells were infected with *H. pylori* alone; and (iii) blank control group, consisting of untreated AGS cells cultured under identical conditions. After incubation, the culture supernatants were collected and centrifuged to remove cellular debris. The concentration of IL-8 in the clarified supernatants was determined using a commercial enzyme-linked immunosorbent assay (ELISA) kit according to the manufacturer’s instructions. The IL-8 assay was performed using culture supernatants collected from a single independent AGS cell experiment. For each treatment, the collected culture supernatant was measured in three technical replicate wells using ELISA. The three technical measurements were averaged to obtain a single IL-8 concentration for each treatment. Because only one independent biological experiment was performed, the IL-8 data were summarized descriptively and were not subjected to inferential statistical analysis.

### 2.17. Zebrafish Prophylactic H. pylori Exposure Model

Wild-type AB zebrafish larvae at 3 days post-fertilization (3 dpf) were used to establish a prophylactic *H. pylori* exposure model. Thirty larvae were allocated to each treatment well of a 6-well plate containing 3 mL of embryo medium. Larvae were pretreated with FP13 or SHY0008 at a final concentration of 1 × 10^8^ CFU/mL, whereas clarithromycin (125 μg/mL) was used as the pharmacological positive control. After 24 h of pretreatment at 28 °C, all groups except the blank control were exposed to CM-DiI-labeled *H. pylori* (5 × 10^7^ CFU/mL) for an additional 24 h. Each treatment group consisted of a single well containing 30 larvae. Therefore, the treatment well, rather than the individual larva, was considered the experimental unit. Individual larvae selected for fluorescence imaging or histopathological assessment were treated as within-well subsamples and not as independent experimental replicates.

Following treatment, six larvae were randomly selected from each group for fluorescence imaging, and intestinal CM-DiI-associated fluorescence intensity was quantified using NIS-Elements D software, version 3.20 (Nikon, Tokyo, Japan). Because these larvae were sampled from the same treatment well, they were considered within-group subsamples rather than independent treatment replicates, and the fluorescence data were interpreted descriptively. Because CM-DiI is a membrane-associated fluorescent dye, the measured fluorescence signal was interpreted as *H. pylori*-associated fluorescent material and not as a direct quantitative measure of viable bacterial colonization or burden. The remaining larvae were not used for quantitative endpoint analyses and were euthanized at the end of the experiment. For histopathological evaluation, three larvae were randomly selected from each treatment well, fixed in 4% paraformaldehyde, paraffin-embedded, sectioned, and stained with hematoxylin and eosin (H&E). Histological scoring was performed on these three larvae. Blinding of the scorer to treatment allocation was not documented. Because only one treatment well was used per group, the treatment well was considered the experimental unit, whereas individual larvae represented within-well subsamples. Accordingly, the histopathological data were summarized descriptively and were not subjected to inferential statistical analysis. The experimental design is summarized in Table 1. Tissue injury was scored according to the criteria provided in Table S9.

### 2.18. Mice H. pylori Infection Model

#### 2.18.1. Animals and Experimental Design

All animal experiments were reviewed and approved by the Laboratory Animal Ethics Committee of Guangdong Huawei Testing Co., Ltd. (Guangzhou, China), with the ethical approval number 20260403. All procedures were conducted in accordance with institutional guidelines and internationally accepted principles for the care and use of laboratory animals. Every effort was made to minimize animal suffering and to reduce the number of animals used. Before animal introduction, the animal facility was disinfected using an individually ventilated cage (IVC) system combined with aerosol disinfectant treatment, and all cages were sterilized by autoclaving. Following a one-week acclimatization period, the mice were housed under SPF conditions at 25 ± 2 °C, 50 ± 10% relative humidity, and a 12 h light/12 h dark cycle, with free access to standard chow and sterile water. A total of 33 female SPF BALB/c mice (6 weeks old) were used in this study. After a one-week acclimatization period, three of the 33 female SPF BALB/c mice were randomly selected and euthanized under anesthesia to confirm the absence of naturally occurring *H. pylori*-associated signals before model establishment. The remaining 30 mice were randomly allocated using a random-number table into five predefined groups (n = 6 per group): blank control, *H. pylori* model, positive-control, FP13, and SHY0008 groups. The blank-control group remained uninfected, whereas the other four groups underwent the *H. pylori* inoculation protocol.

In a preliminary assessment, culture-based detection of gastric *H. pylori* was affected by background microbial growth and did not permit reliable selective identification or enumeration. Therefore, direct culture-based verification of infection in individual mice was not performed before intervention.

For model establishment, mice received amoxicillin (100 mg/kg) and metronidazole (40 mg/kg) by oral gavage once daily for three consecutive days. After fasting for 12 h, each mouse received 0.2 mL of 5% NaHCO_3_, followed 1 h later by 0.5 mL of *H. pylori* ATCC 43504 suspension (1 × 10^8^ CFU/mL) once daily for 14 consecutive days. After completion of the inoculation period, the predefined interventions were initiated according to the original group allocation. *H. pylori*-associated infection status was subsequently evaluated at the experimental endpoint using gastric *H. pylori* antigen levels, gastric urease activity, and histopathological findings.

**Figure 1:**
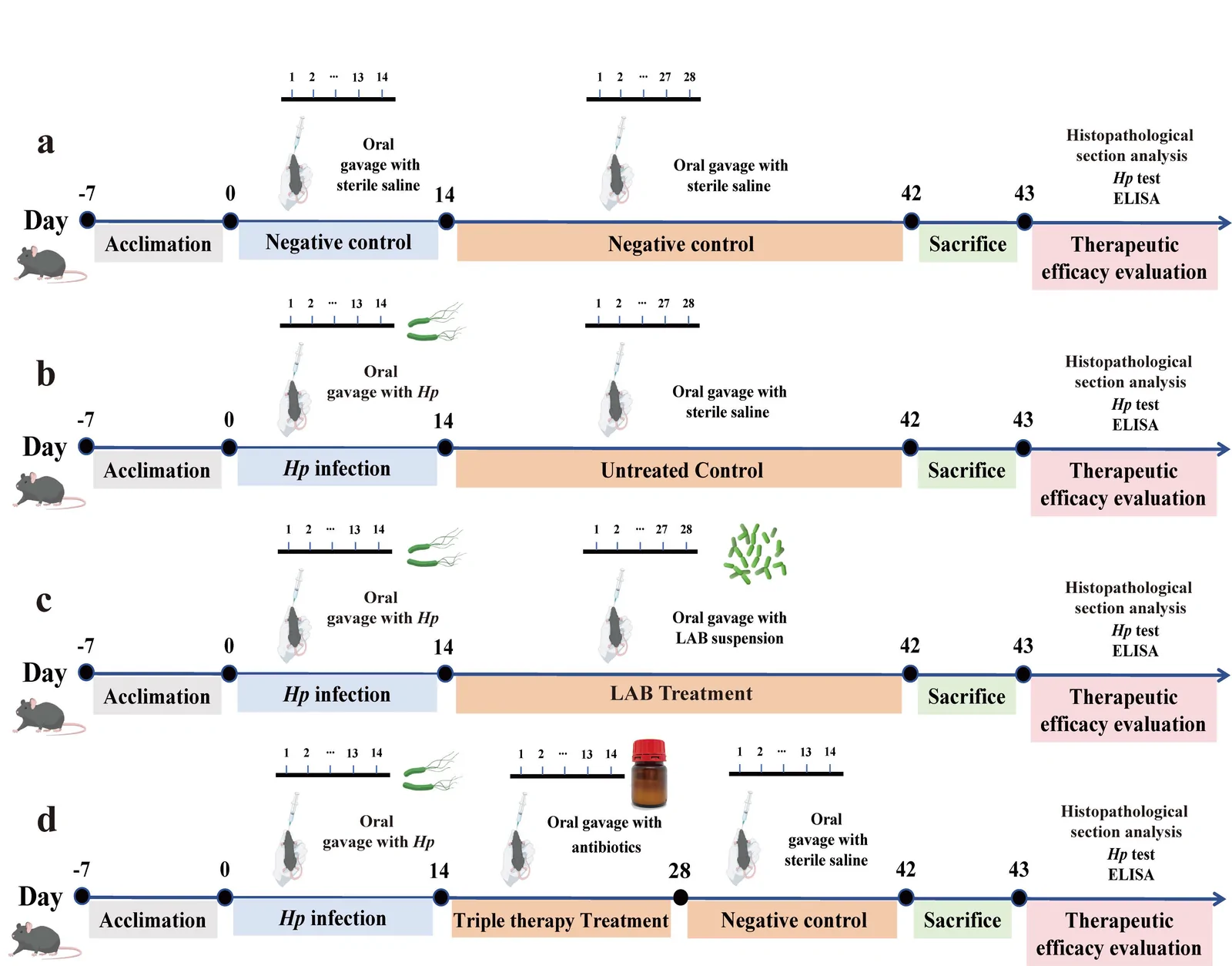
Experimental design of the probiotic intervention in the *H. pylori*-infected mouse model. (**a**) Blank control group. (**b**) *H. pylori* model group. (**c**) Probiotic treatment group. (**d**) Positive control group. Mice received triple therapy consisting of omeprazole (5.2 mg/kg/day), amoxicillin (260 mg/kg/day), and clarithromycin (65 mg/kg/day) by oral gavage.

The experimental protocol is illustrated in Figure 1, and the treatment regimen is summarized in Table 2.

Body weight was recorded weekly throughout the experimental period to monitor the growth status of the mice. At the end of the experiment, mice were fasted overnight (approximately 12 h) with free access to water before sample collection. Blood samples were collected from the retro-orbital venous plexus under isoflurane anesthesia and transferred into serum-separation tubes. After standing at room temperature for 1 h to allow clot formation, the samples were centrifuged at 2000× *g* for 10 min. The resulting serum was collected and stored at −80 °C until subsequent analysis. Following blood collection, mice were euthanized under isoflurane anesthesia. The abdominal cavity was immediately opened, and the pyloric sphincter was ligated to prevent leakage of gastric contents. Subsequently, 500 μL of sterile PBS was gently injected into the stomach through the esophagus. The stomach was carefully massaged, and the gastric lavage fluid was collected for subsequent analysis. The stomach was then excised, opened longitudinally along the greater curvature, and divided into three portions. One portion was homogenized for the determination of *H. pylori* urease activity. A second portion was fixed in 4% paraformaldehyde for histopathological examination. The remaining portion was snap-frozen in liquid nitrogen immediately after collection and stored at −80 °C for subsequent molecular analyses.

Personnel responsible for treatment administration were aware of group allocation because the interventions differed in composition. For gastric *H. pylori* antigen ELISA, serum CXCL1/KC ELISA, and gastric urease measurements, samples were coded before analysis so that investigators performing these assays were unaware of treatment identity. Histopathological assessment was performed by a pathologist blinded to group allocation. Formal allocation concealment was not implemented. No animals died during the experiment, and no animals or data points were excluded from the final analysis.

#### 2.18.2. Determination of *H. pylori* Antigen Levels

The level of gastric *H. pylori* whole-cell antigen was quantified using a double-antibody sandwich ELISA. Gastric lavage fluid samples were centrifuged at 12,000× *g* for 10 min at 4 °C, and the supernatants were collected for analysis. The antigen concentration was determined using a Mouse *H. pylori* Antigen ELISA Kit (ZeYe Biotechnology Co., Ltd., Shanghai, China) according to the manufacturer’s instructions. The assay microplate was pre-coated with *H. pylori*-specific polyclonal antibodies capable of recognizing whole-cell (total bacterial) antigens. Following incubation, washing, enzyme conjugation, substrate development, and reaction termination according to the manufacturer’s protocol, the absorbance of each well was measured at 450 nm using a microplate reader. Antigen concentrations were calculated from the corresponding standard curve and expressed as ng/L. The measured antigen level was used as an indirect indicator of gastric *H. pylori*-associated bacterial material and was not interpreted as evidence of viable bacterial colonization or as a direct measure of viable bacterial burden.

#### 2.18.3. Determination of Gastric Urease Activity

Gastric urease activity was determined using a modified phenol red assay. A portion of the gastric antral tissue was homogenized using a high-speed refrigerated tissue homogenizer. The homogenate was transferred into a 96-well microplate and thoroughly mixed with the urease indicator solution. After incubation at 37 °C for 30 min, the color change was visually observed, and the absorbance was measured at 561 nm using a multimode microplate reader. Gastric urease activity was interpreted as an *H. pylori*-associated functional surrogate marker and not as a direct quantitative measure of viable gastric *H. pylori*.

#### 2.18.4. Determination of Serum CXCL1/KC Levels by ELISA

Serum CXCL1/KC concentrations were measured using a QuantiCyto^®^ Mouse KC (IL-8) ELISA Kit (NeoBioscience Technology Co., Ltd., Shenzhen, China; Cat. No. EMC104.48) according to the manufacturer’s instructions. The assay is specific for murine KC/CXCL1, with a sensitivity of 7.8 pg/mL and intra- and inter-assay coefficients of variation below 10%, and does not cross-react with human IL-8 or rat IL-8. Serum samples from three individual mice per group were analyzed, and absorbance was measured at 450 nm. CXCL1/KC concentrations were calculated from the standard curve and expressed as pg/mL.

#### 2.18.5. Histopathological Examination of Gastric Tissue

Gastric tissues fixed in 4% paraformaldehyde were dehydrated, embedded in paraffin, and sectioned at a thickness of 4 μm. The sections were subsequently stained with H&E according to standard histological procedures. Histopathological changes were examined and scored by a pathologist blinded to treatment-group allocation. Gastric mucosal injury and inflammatory cell infiltration were evaluated using a standardized histopathological scoring system. The detailed scoring criteria are provided in Table S10.

### 2.19. Whole-Genome Sequencing and Assembly

Genomic DNA of *Lactiplantibacillus plantarum* SHY0008 was subjected to whole-genome sequencing using Illumina short-read and Oxford Nanopore long-read sequencing. For short-read sequencing, libraries were quality-checked using a Qubit fluorometer (Thermo Fisher Scientific, Waltham, MA, USA) and an Agilent 2100 Bioanalyzer(Agilent Technologies, Santa Clara, CA, USA) and were subsequently sequenced on an Illumina platform using a paired-end 150-bp (PE150) strategy. For SHY0008, 21,055,470 reads were generated for each of the R1 and R2 datasets, yielding 6,316,641,000 bp of raw sequence data in total. The proportions of bases with quality scores above Q30 were 97.81% for R1 and 97.63% for R2.

Raw Illumina reads were quality-filtered using Trimmomatic v0.38 with the parameters ILLUMINACLIP:TruSeq3-PE.fa:2:30:10:8:true, AVGQUAL:15, LEADING:5, TRAILING:5, and MINLEN:80. Raw and cleaned data were evaluated using MultiQCv1.31, and sequencing output was summarized using ReSeqTools (https://github.com/hewm2008/Reseqtools, accessed on 30 July 2026). After quality filtering, 42,094,498 clean reads comprising 6,244,374,768 bp were retained, corresponding to 6.24 Gb of clean data and an estimated short-read sequencing depth of approximately 1690× based on the final draft genome size.

Oxford Nanopore long reads were also used together with the Illumina short reads for hybrid genome assembly. According to the retained sequencing records, long-read sequencing was performed using a PromethION R10.4.1 flow cell (Oxford Nanopore Technologies, Oxford, UK). Detailed run-level information regarding the Nanopore basecalling software and long-read quality statistics was not available in the retained project records and was therefore not inferred. Hybrid assembly was performed using Unicycler v0.5.1. Assembly quality was evaluated using CheckM v1.2.4. However, the numerical completeness and contamination outputs were not available in the retained project records and were therefore not retrospectively inferred. Assembly statistics, including genome size, GC content, contig number, N50, L50, and largest contig size, were reported directly from the retained assembly records.

### 2.20. Genome-Based Taxonomic Identification

Genome-based taxonomic identification of SHY0008 was performed using average nucleotide identity (ANI) analysis with the Species Identification module of ProbioMinServer2. The SHY0008 draft genome was compared with representative *Lactiplantibacillus plantarum* genomes, including the type strain *L. plantarum* subsp. plantarum ATCC 14917. ANI values were calculated for each genome comparison, and a species-level ANI threshold of approximately 95–96% was used for taxonomic assignment. The reference strains, genome assembly accessions, and corresponding ANI values are provided in Table 3.

### 2.21. Genomic Functional Annotation Analysis

The protein sequences of predicted coding genes were annotated against the Kyoto Encyclopedia of Genes and Genomes (KEGG) and Cluster of Orthologous Groups of proteins (COG) databases using EggNOG-mapper v2. The functional classification of annotated genes was performed based on their orthologous groups and associated metabolic pathways. In addition, secondary metabolite biosynthetic gene clusters were identified using antiSMASH v8.0.4, which was employed to predict potential biosynthetic pathways, including bacteriocin and other antimicrobial compound-associated gene clusters.

### 2.22. Preliminary Genome-Based Safety Screening

The SHY0008 draft genome was subjected to preliminary genome-based safety screening using the Safety Analysis module of ProbioMinServer2. Acquired antimicrobial resistance genes were screened using CARD, ResFinder, and AMRFinderPlus, whereas potential virulence-associated genes were evaluated using VFDB and VirulenceFinder. Default parameters provided by the server were used. These analyses were used as a preliminary screen for known acquired antimicrobial resistance and virulence-associated determinants and were not considered a comprehensive evaluation of probiotic safety.

### 2.23. Statistical Analysis

Statistical analyses were performed using GraphPad Prism version 10.2. Independent biological replicates were treated as the experimental units for inferential statistical analysis. When multiple technical measurements were obtained from the same biological replicate, technical replicates were averaged before statistical analysis and were not treated as independent observations. Data derived solely from technical replicates or subsamples within a single biological preparation or treatment condition were summarized descriptively and were not subjected to inferential statistical testing. The numbers of biological and technical replicates for individual assays are specified in the corresponding Methods sections and figure legends. Data are presented as mean ± SD unless otherwise stated. A *p* value < 0.05 was considered statistically significant.

Longitudinal mouse body-weight data were analyzed using two-way repeated-measures ANOVA, with treatment group and time as factors and with assessment of the treatment × time interaction, followed by Tukey’s multiple-comparison test where appropriate. In contrast, the auto-aggregation and mixed-suspension aggregation time-course data were obtained from technical replicate wells derived from a single bacterial preparation and therefore lacked independent biological replication. These aggregation kinetics were consequently summarized descriptively and were not subjected to repeated-measures or other inferential statistical analyses.

## 3. Results

### 3.1. Isolation, Preliminary Screening, and Identification of SHY0008

**Figure 2:**
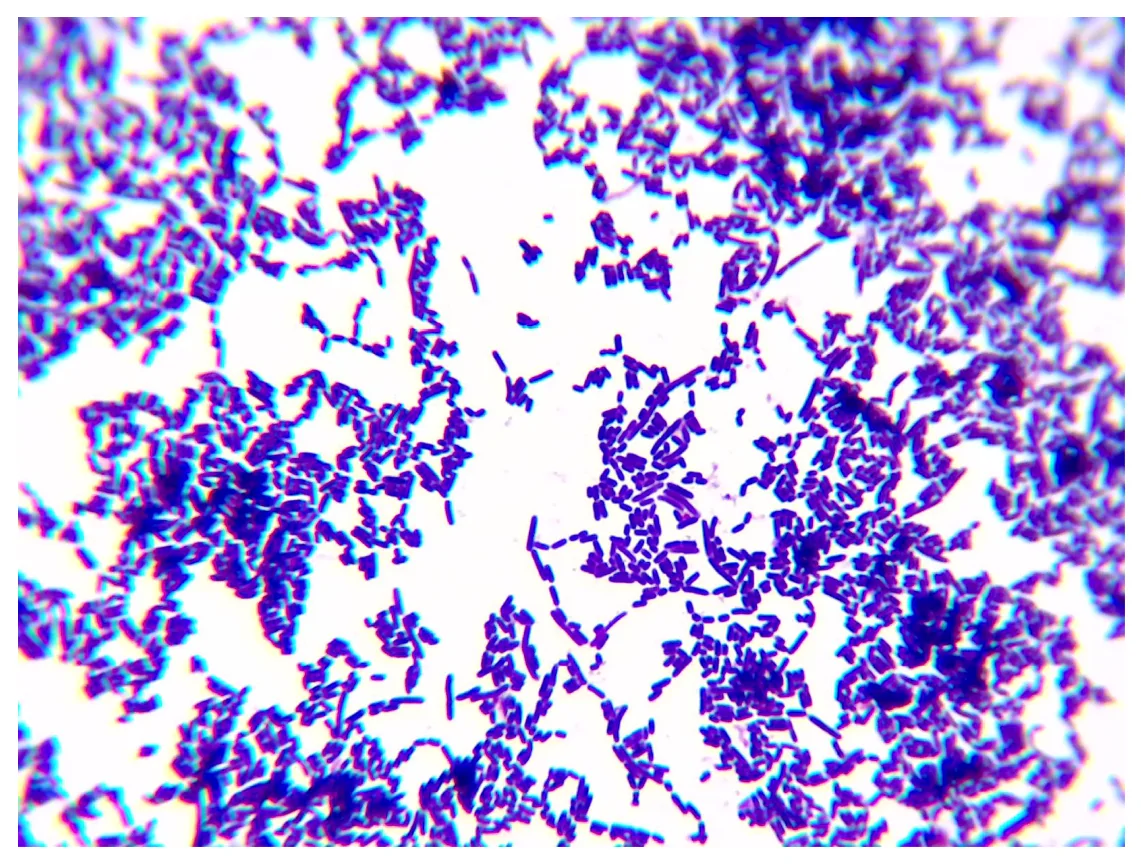
Gram staining of *Lactiplantibacillus plantarum* SHY0008 at 1000× magnification.

A total of 12 Gram-positive and catalase-negative LAB isolates, designated SHY0001–SHY0012, were recovered from Miao sour soup and subjected to preliminary screening based on inhibition of *H. pylori* urease activity. SHY0008 showed the highest mean urease inhibition. Based on this preliminary screening result, SHY0008 was selected for subsequent comprehensive evaluation. The screening results for all isolates are provided in Table S7. Subsequent 16S rRNA gene sequence analysis identified the isolate as *Lactiplantibacillus plantarum* SHY0008. As shown in Figure 2, Gram staining revealed that SHY0008 exhibited a purple-stained, short rod-shaped morphology, occurring either singly or in short chains, which is consistent with the typical morphological characteristics of *Lactiplantibacillus plantarum*.

Preliminary 16S rRNA gene sequence analysis assigned SHY0008 to the *Lactiplantibacillus plantarum* group. Because 16S rRNA sequencing alone provides limited discriminatory resolution among closely related members of this group, species-level identification was further evaluated using whole-genome ANI analysis. SHY0008 showed an ANI of 98.22% against the *Lactiplantibacillus plantarum* type strain *Lactiplantibacillus plantarum* subsp. plantarum ATCC 14917 (GenBank assembly accession GCA_000143745.1), which is well above the commonly accepted species-level ANI threshold of approximately 95–96%. Comparisons with additional *L. plantarum* reference assemblies yielded ANI values of 98.18–98.22%. Together, these genome-based results support the identification of SHY0008 as *Lactiplantibacillus plantarum*.

### 3.2. Acid and Bile Salt Tolerance of Lactiplantibacillus plantarum SHY0008

**Figure 3:**
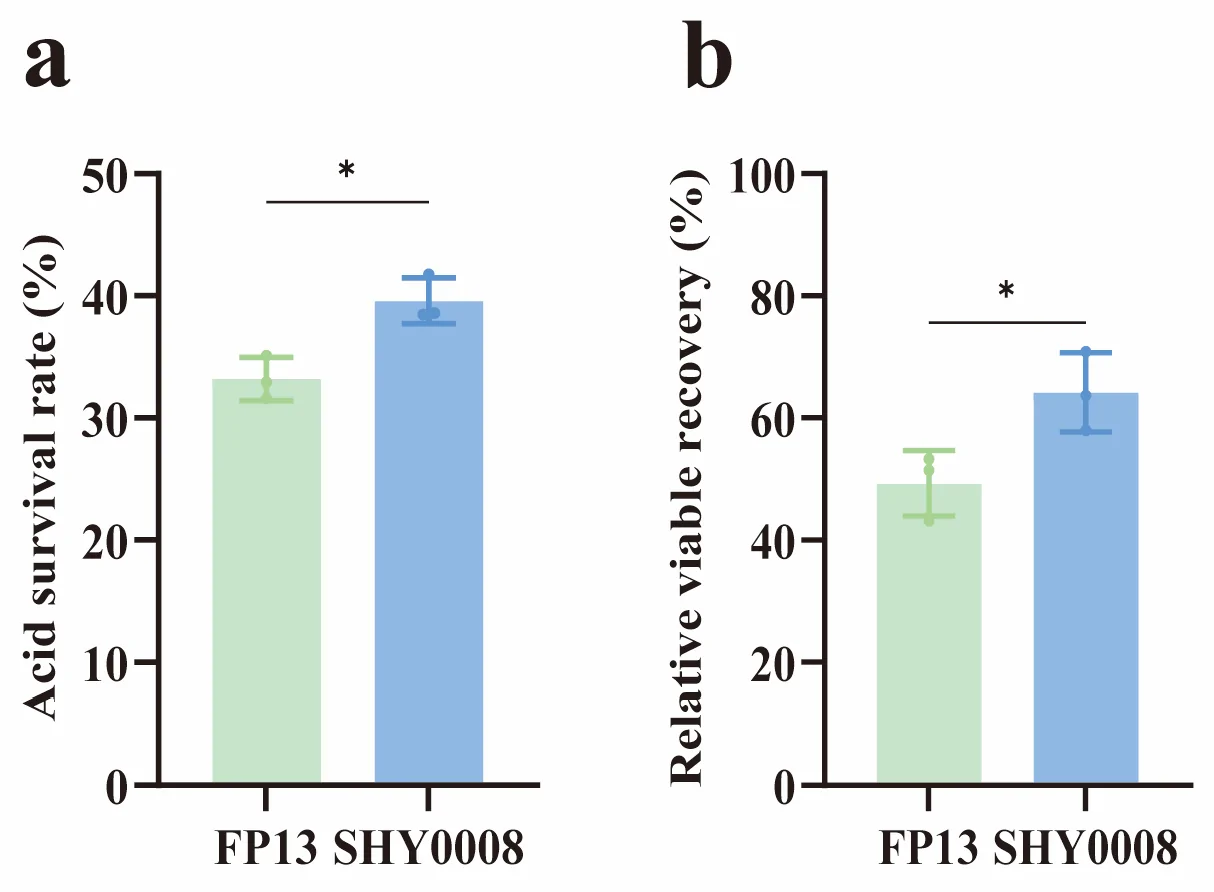
Tolerance of FP13 and SHY0008 under simulated gastrointestinal conditions. (**a**) Survival rates after 4 h of exposure to artificial gastric juice initially adjusted to pH 2.0; the measured final pH values after mixing were 2.39 ± 0.03 for FP13 and 2.39 ± 0.02 for SHY0008. (**b**) Relative viable recovery after exposure to 0.3% (*w*/*v*) oxgall bile salts for 4 h. Data are presented as mean ± SD from three independent biological replicates per strain (n = 3), with each biological replicate quantified using three technical replicate agar plates. Technical replicate plate counts were averaged before statistical analysis. * *p* < 0.05.

The tolerance of FP13 and SHY0008 to simulated gastrointestinal conditions was evaluated using three independent biological replicates per strain. The artificial gastric juice was initially adjusted to pH 2.0, whereas the measured final pH values after mixing with the bacterial suspensions were 2.39 ± 0.03 for FP13 and 2.39 ± 0.02 for SHY0008. After 4 h of exposure, the survival rates of FP13 and SHY0008 were 33.22 ± 1.78% and 39.61 ± 1.87%, respectively (Figure 3a), with SHY0008 showing significantly higher survival than FP13 under the tested acidic conditions (*p* < 0.05).

### 3.3. In Vitro Probiotic Properties of Lactiplantibacillus plantarum SHY0008

**Figure 4:**
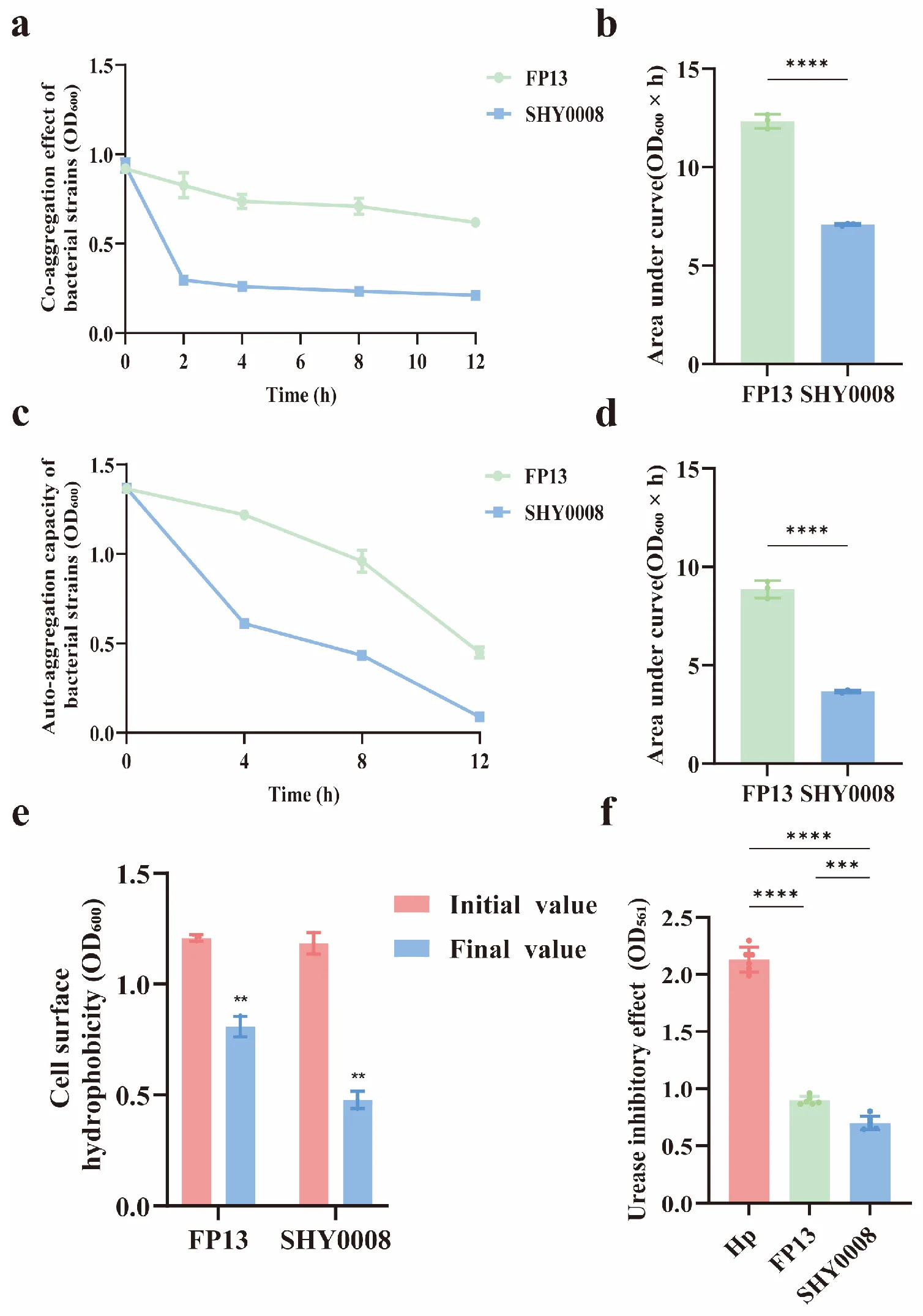
Physiological characteristics and in vitro anti-*H. pylori* activities of SHY0008. (**a**,**b**) Auto-aggregation kinetics and AUC; (**c**,**d**) mixed-suspension aggregation kinetics with *H. pylori* and AUC; (**e**) cell surface hydrophobicity; (**f**) urease-associated phenol-red response of *H. pylori* following FP13 or SHY0008 treatment, with or without pH adjustment to 6.5. (**a**–**d**) are descriptive data from technical replicates of a single bacterial preparation and were not subjected to inferential statistical analysis. Panels (**e**,**f**) represent three independent biological replicates (n = 3) and were analyzed using an unpaired two-tailed t-test. Statistical significance is indicated as ** *p* < 0.01, *** *p* < 0.001 and **** *p* < 0.0001.

The auto-aggregation and mixed-suspension aggregation characteristics of FP13 and SHY0008 were evaluated by monitoring changes in OD600 during static incubation. In the auto-aggregation assay, the OD600 values of both strains progressively decreased over 12 h, indicating bacterial aggregation and sedimentation. At 12 h, the observed auto-aggregation rates were 67.06 ± 2.99% for FP13 and 93.47 ± 0.15% for SHY0008 (Figure 4a). The corresponding AUC values of the OD600-time curves were 12.34 ± 0.36 and 7.09 ± 0.06, respectively (Figure 4b). The lower observed AUC for SHY0008 was consistent with a more rapid decrease in suspended bacterial cells under the tested conditions. During co-incubation with *H. pylori*, both strains also exhibited time-dependent decreases in OD600. At 12 h, the observed mixed-suspension aggregation rates were 32.61 ± 0.95% for FP13 and 77.35 ± 0.44% for SHY0008 (Figure 4c). The corresponding AUC values were 8.86 ± 0.44 and 3.67 ± 0.08, respectively (Figure 4d). Under the conditions of this experiment, SHY0008 showed a higher observed mixed-suspension aggregation level and a lower OD600-time AUC than FP13. Because the auto-aggregation and mixed-suspension aggregation measurements were based on three technical replicate wells derived from a single bacterial preparation, these data were interpreted descriptively and were not subjected to inferential statistical testing. Cell surface hydrophobicity was further evaluated using three independent biological replicates. The hydrophobicity values of FP13 and SHY0008 were 36.48 ± 3.72% and 60.21 ± 2.71%, respectively (Figure 4e). SHY0008 exhibited significantly higher cell surface hydrophobicity than FP13.

### 3.4. Effects of FP13 and SHY0008 on the H. pylori Urease-Associated Phenol-Red Response

The urease-associated phenol-red response was evaluated in three independent biological experiments. The mean OD561 value of the *H. pylori* control was 2.234 ± 0.201, whereas the corresponding values were 1.410 ± 0.076 for FP13 and 1.017 ± 0.003 for SHY0008, representing reductions of 36.62 ± 5.71% and 54.23 ± 3.98%, respectively. To evaluate the contribution of probiotic-associated acidification to the colorimetric response, additional co-cultures were adjusted to pH 6.5 before measurement. After pH adjustment, the OD561 values were 1.557 ± 0.042 for FP13 and 1.142 ± 0.041 for SHY0008, corresponding to reductions of 29.88 ± 6.98% and 48.72 ± 2.88% relative to the *H. pylori* control. Both pH-adjusted groups remained significantly lower than the *H. pylori* control (*p* < 0.001), whereas pH adjustment produced only modest numerical increases relative to the corresponding untreated probiotic groups. These findings indicate that acidification may contribute to the reduction in the phenol-red response but do not fully account for the observed effect. Because this colorimetric assay is pH sensitive, the results are interpreted as changes in the urease-associated phenol-red response rather than definitive evidence of direct enzymatic inhibition.

### 3.5. Anti-H. pylori Activity of Lactiplantibacillus plantarum SHY0008

#### 3.5.1. Inhibitory Effects of FP13 and SHY0008 Against *H. pylori* in the Agar Well Diffusion Assay

**Figure 5:**
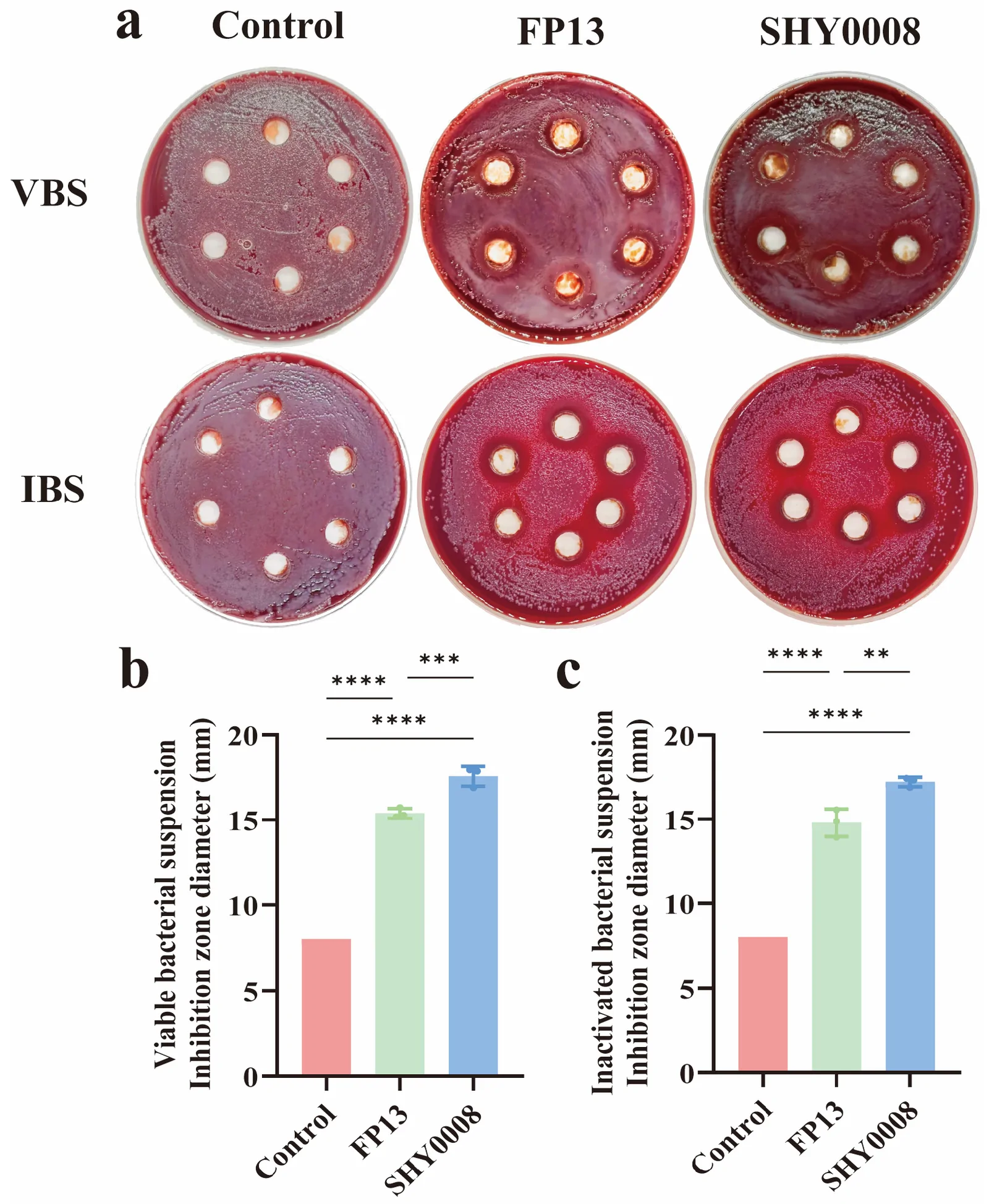
Inhibitory effects of FP13 and SHY0008 against *H. pylori* in the Oxford cup diffusion assay. (**a**) Representative inhibition zones produced by VBS and HTSBP. (**b**,**c**) Quantification of inhibition zone diameters. Data are presented as mean ± SD from three independent biological replicates (n = 3), each derived from an independently prepared bacterial culture. Statistical significance is indicated as ** *p* < 0.01, *** *p* < 0.001 and **** *p* < 0.0001.

As shown in Figure 5 and Table 4, the sterile PBS vehicle control showed no inhibition, with an apparent diameter of 8.00 ± 0.00 mm corresponding to the inner diameter of the Oxford cup. FP13 and SHY0008 viable bacterial suspensions (VBS) produced inhibition zones of 15.39 ± 0.29 and 17.56 ± 0.58 mm, respectively, whereas the corresponding heat-treated and sonicated bacterial preparations (HTSBPs) produced zones of 14.79 ± 0.81 and 17.21 ± 0.29 mm. SHY0008 produced larger inhibition zones than FP13 under both preparation conditions. Because HTSBPs contained heat-treated and sonication-disrupted bacterial material, their activity was not attributed to intact non-viable cells or to a specific heat-stable component.

#### 3.5.2. Inhibitory Effects of CFS from *Lactiplantibacillus plantarum* SHY0008 on *H. pylori* Growth

**Figure 6:**
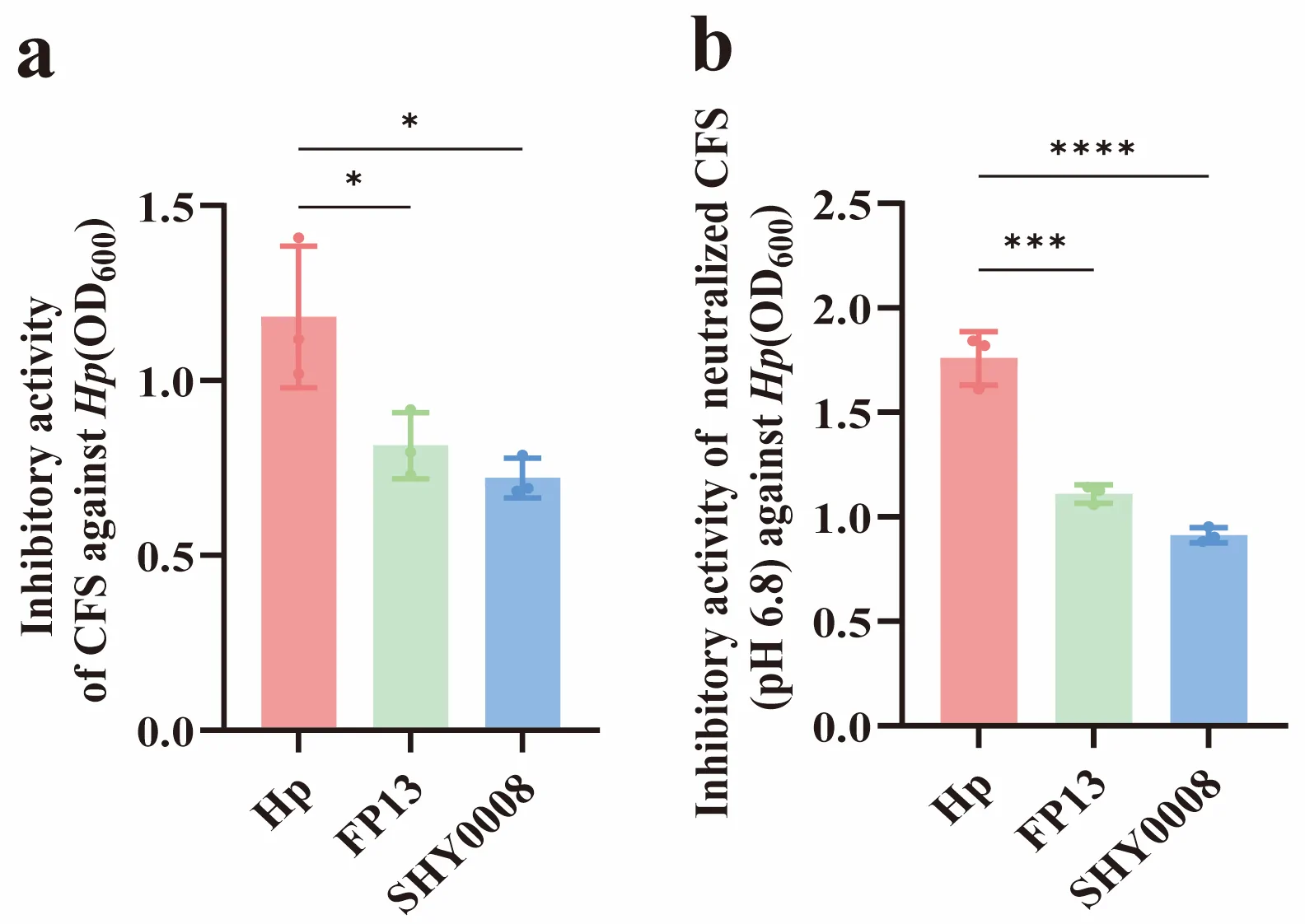
Inhibitory effects of CFSs from FP13 and SHY0008 against *H. pylori* growth. Inhibitory effects of (**a**) NCFS and (**b**) PACFS from FP13 and SHY0008 on *H. pylori* growth. Data are presented as mean ± SD from three independent biological replicates (n = 3), with each replicate measured in triplicate technical wells. Technical replicate measurements were averaged before statistical analysis. Statistical significance is indicated as * *p* < 0.05, *** *p* < 0.001 and **** *p* < 0.0001.

The inhibitory effects of CFS derived from FP13 and SHY0008 on *H. pylori* culture optical density were evaluated using three independent biological replicates, each measured in triplicate technical wells. Both native CFS (NCFS) preparations reduced *H. pylori* OD600 relative to the untreated control, with SHY0008 showing the greater reduction (Table 5; Figure 6). After adjustment of the CFS to pH 6.8, inhibitory activity was partially retained, and SHY0008 again showed a greater OD600 reduction than FP13. These findings indicate that low pH alone did not account for the entire observed effect.

#### 3.5.3. Effects of CFS on *H. pylori* Viability-Associated Fluorescence

**Figure 7:**
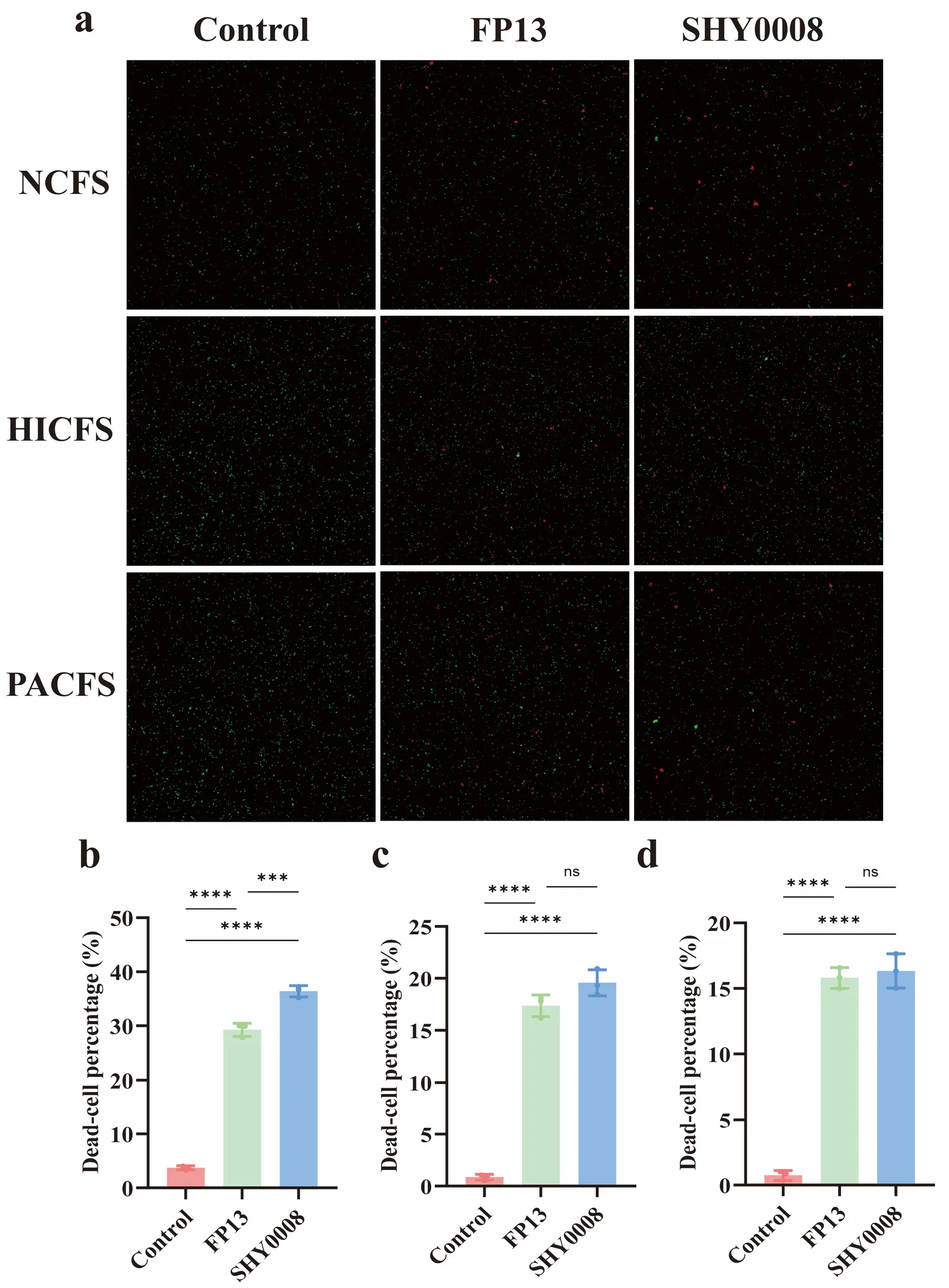
Effects of FP13- and SHY0008-derived CFSs on *H. pylori* viability. (**a**) Representative LIVE/DEAD fluorescence images. (**b**–**d**) Instrument-derived dead-cell percentages after NCFS, HICFS, and PACFS treatment, respectively. Data are presented as mean ± SD from three independent biological replicates (n = 3). Data are presented as the mean ± SD. Statistical significance is indicated as ns *p* > 0.05, *** *p* < 0.001 and **** *p* < 0.0001.

To further evaluate the effects of CFS on *H. pylori* membrane-integrity-associated fluorescence, LIVE/DEAD staining was performed after CFS exposure (Figure 7). As shown in Table 6, the instrument-derived dead-cell percentages were 29.27 ± 1.19% and 36.38 ± 1.04% following FP13-NCFS and SHY0008-NCFS treatment, respectively, compared with 3.73 ± 0.37% in the corresponding control. HICFS treatment yielded values of 17.36 ± 1.04% for FP13 and 19.57 ± 1.25% for SHY0008, whereas PACFS treatment yielded 15.80 ± 0.80% and 16.33 ± 1.31%, respectively. Overall, CFS exposure increased the proportion of *H. pylori* cells exhibiting dead-cell-associated fluorescence, with the largest increase observed after SHY0008-NCFS exposure. These fluorescence-based findings were not interpreted as equivalent to CFU-based bacterial killing. The assay was independently performed three times using independently prepared CFS samples and *H. pylori* cultures (n = 3 biological replicates).

**Table 6:** Effects of CFS treatment on *H. pylori* dead-cell-associated fluorescence percentage.

Strain	Dead-Cell Percentage of *H. pylori* Treated with NCFS (%)	Dead-Cell Percentage of *H. pylori* Treated with HICFS (%)	Dead-Cell Percentage of *H. pylori* Treated with PACFS (%)
Control	3.73 ± 0.37	0.87 ± 0.27	0.73 ± 0.37
FP13	29.27 ± 1.19	17.36 ± 1.04	15.80 ± 0.80
SHY0008	36.38 ± 1.04	19.57 ± 1.25	16.33 ± 1.31

### 3.6. Lactiplantibacillus plantarum SHY0008 Enhances Gastric Epithelial Cell Adhesion and Attenuates H. pylori-Induced Inflammatory Responses

**Figure 8:**
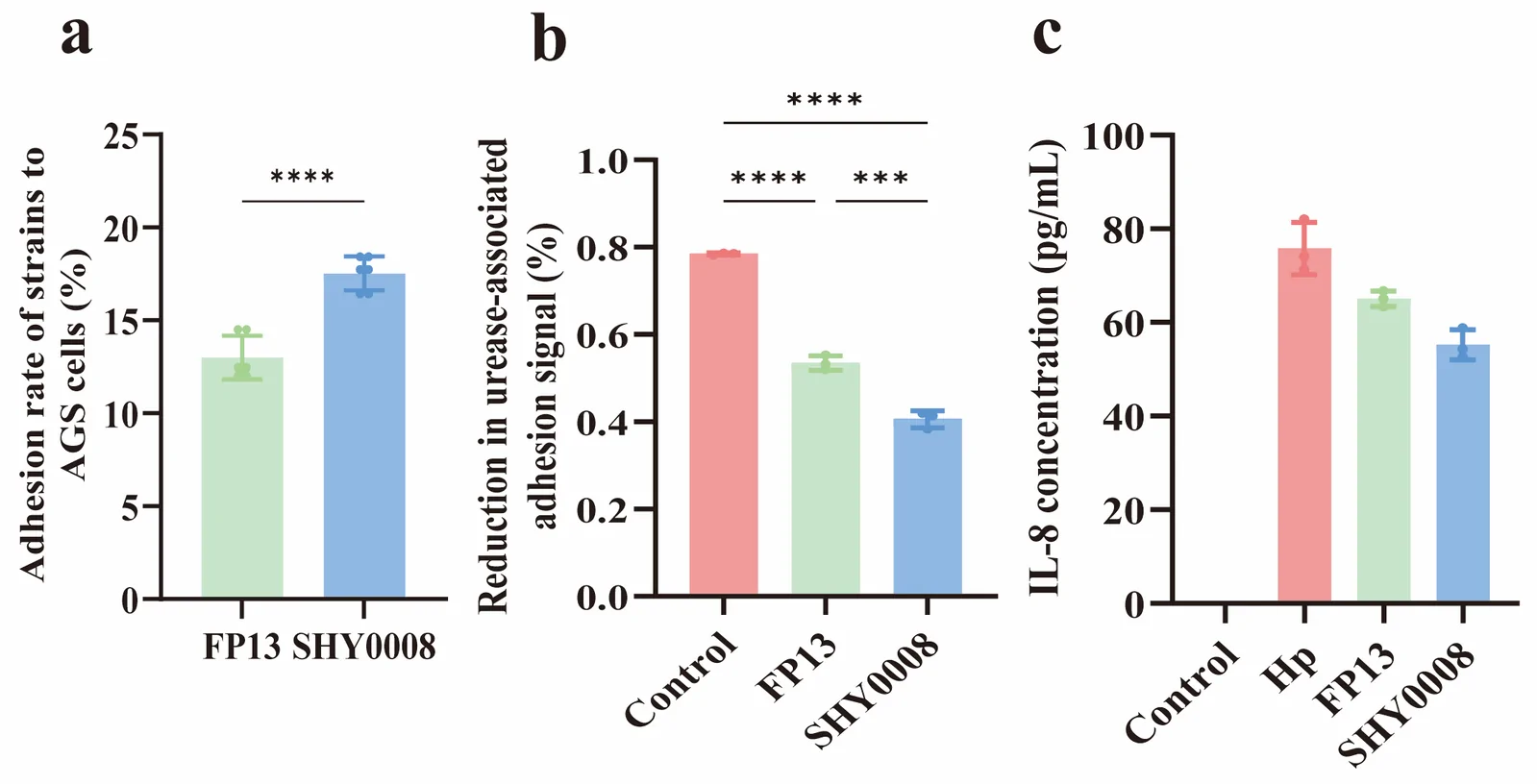
Anti-*H. pylori* activity of FP13 and SHY0008 in the AGS cell model. (**a**) Adhesion of FP13 and SHY0008 to AGS cells; (**b**) relative reduction in the urease-associated *H. pylori* adhesion signal; and (**c**) IL-8 secretion in *H. pylori*-infected AGS cells. For panels (**a**,**b**), data are mean ± SD from three independent biological replicates (n = 3), each measured in triplicate technical replicates. Panel (**c**) shows mean ± SD of three technical ELISA wells from a single biological experiment and is descriptive only. Data are presented as the mean ± SD. Statistical significance for panels (**a**,**b**) is indicated as *** *p* < 0.001 and **** *p* < 0.0001.

AGS cells, a widely used human gastric epithelial cell model, were applied to evaluate the adhesion capacity of probiotic strains and their potential protective effects against *H. pylori* infection. The adhesion abilities of FP13 and SHY0008 to AGS cells were first evaluated in three independent biological experiments. The adhesion rates were 13.00 ± 1.32% for FP13 and 17.53 ± 1.01% for SHY0008 (Figure 8a), indicating a higher adhesion capacity of SHY0008 under the tested conditions.

The effects of FP13 and SHY0008 on cell-associated *H. pylori* were subsequently assessed using the urease-based colorimetric assay. The relative reductions in the urease-associated adhesion signal were 31.85 ± 2.09% for FP13 and 48.27 ± 2.52% for SHY0008, respectively (Figure 8b). SHY0008 produced a greater reduction in the urease-associated signal than FP13 under the tested conditions. Because this assay relies on residual urease activity after removal of non-adherent bacteria, these values represent indirect estimates of *H. pylori* adhesion rather than direct viable bacterial counts.

IL-8 secretion was further measured in culture supernatants from a single AGS cell experiment. *H. pylori* infection was associated with increased IL-8 secretion, whereas treatment with FP13 or SHY0008 was associated with lower IL-8 levels. As shown in Table 7, the observed IL-8 concentrations were 75.79 ± 5.57 pg/mL in the *H. pylori* group, 65.05 ± 1.70 pg/mL in the FP13 group, and 55.24 ± 3.21 pg/mL in the SHY0008 group (Figure 8c). Because these values were derived from three technical ELISA wells of a single biological experiment, the IL-8 results were interpreted descriptively and were not subjected to inferential statistical analysis.

**Table 7:** Effects of FP13 and SHY0008 on IL-8 secretion in *H. pylori*-infected AGS cells.

Group	IL-8 Concentration (pg/mL)
Blank control	0
*H. pylori* control	75.79 ± 5.57
FP13	65.05 ± 1.70
SHY0008	55.24 ± 3.21

Values are presented as mean ± SD of three technical ELISA wells from a single biological experiment. The data are descriptive only and were not subjected to inferential statistical analysis.

### 3.7. Prophylactic Effects of Clarithromycin, FP13, and SHY0008 in the Zebrafish H. pylori Exposure Model

**Figure 9:**
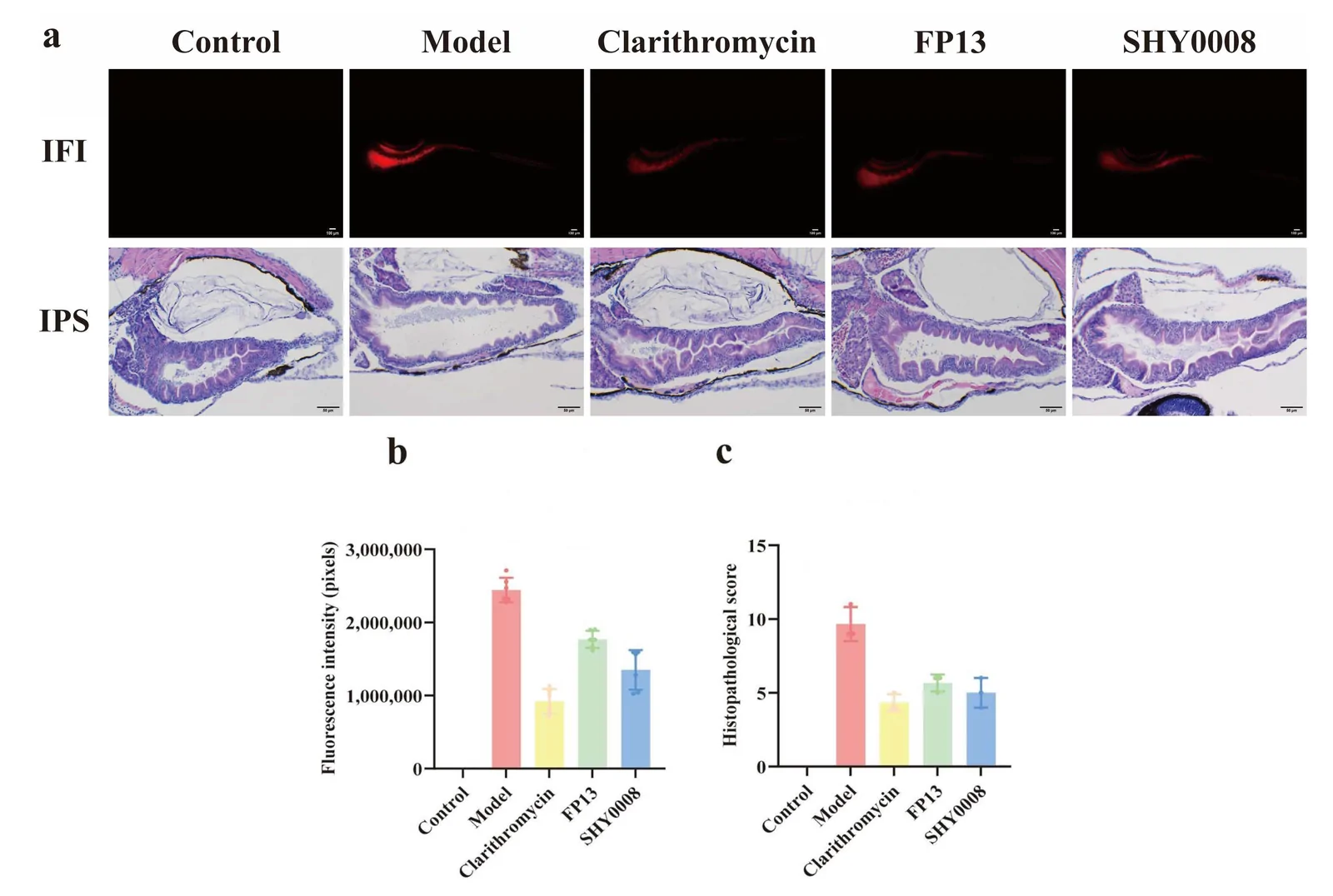
Effects of clarithromycin, FP13, and SHY0008 in the zebrafish *H. pylori* exposure model. (**a**) Representative fluorescence images and H&E-stained intestinal sections from the blank control, *H. pylori* model, clarithromycin, FP13, and SHY0008 groups. (**b**) Quantification of intestinal CM-DiI-associated fluorescence intensity from six larvae per group. (**c**) Histopathological evaluation based on three larvae per group. Because larvae within each group were sampled from the same treatment well, the fluorescence and histopathological data are presented descriptively and were not subjected to inferential statistical testing. CM-DiI-associated fluorescence was used as an indirect indicator of *H. pylori*-associated bacterial material and was not interpreted as a direct measure of viable bacterial colonization.

To evaluate the prophylactic protective effects of FP13 and SHY0008 against subsequent *H. pylori* exposure in vivo, a zebrafish pretreatment model was established, with clarithromycin included as the pharmacological positive control (Figure 9). Strong intestinal CM-DiI-associated fluorescence was observed in the *H. pylori* model group, with a mean fluorescence intensity of 2,444,394 ± 167,141 pixels. Clarithromycin treatment produced the lowest fluorescence signal among the intervention groups, with a mean intensity of 921,190 ± 166,799 pixels. Lower fluorescence signals were also observed in the FP13- and SHY0008-treated groups, with mean intensities of 1,768,294 ± 117,505 and 1,350,455 ± 271,372 pixels, respectively. Compared with the model group, the observed fluorescence intensity was reduced by 62.31% in the clarithromycin group, 27.66% in the FP13 group, and 44.75% in the SHY0008 group. Thus, clarithromycin produced the greatest overall reduction in intestinal CM-DiI-associated fluorescence, whereas SHY0008 showed the lowest observed fluorescence intensity among the probiotic-treated groups. Because the six quantified larvae within each group were sampled from a single treatment well, these fluorescence data were interpreted descriptively and were not subjected to inferential statistical analysis.

Histopathological examination was performed using three larvae from each group. The blank control group showed intact intestinal architecture and had a median histopathological score of 0 (range, 0–0). In contrast, the *H. pylori* model group exhibited pronounced intestinal injury, including shortened, sparse, fused, and flattened mucosal folds, enlargement of the intestinal lumen, and thinning of the intestinal wall, with a median histopathological score of 9 (range, 9–11). Clarithromycin markedly ameliorated these pathological changes and showed the lowest histopathological score among the intervention groups, with a median of 4 (range, 4–5). FP13 and SHY0008 treatment were also associated with improved intestinal morphology, with median scores of 6 (range, 5–6) and 5 (range, 4–6), respectively. Thus, SHY0008 showed a lower observed histopathological score than FP13, whereas clarithromycin produced the greatest overall histopathological improvement. These histopathological observations were interpreted descriptively.

### 3.8. Lactiplantibacillus plantarum SHY0008 Reduces Gastric H. pylori-Associated Markers and Alleviates Gastric Mucosal Inflammation in Mice

**Figure 10:**
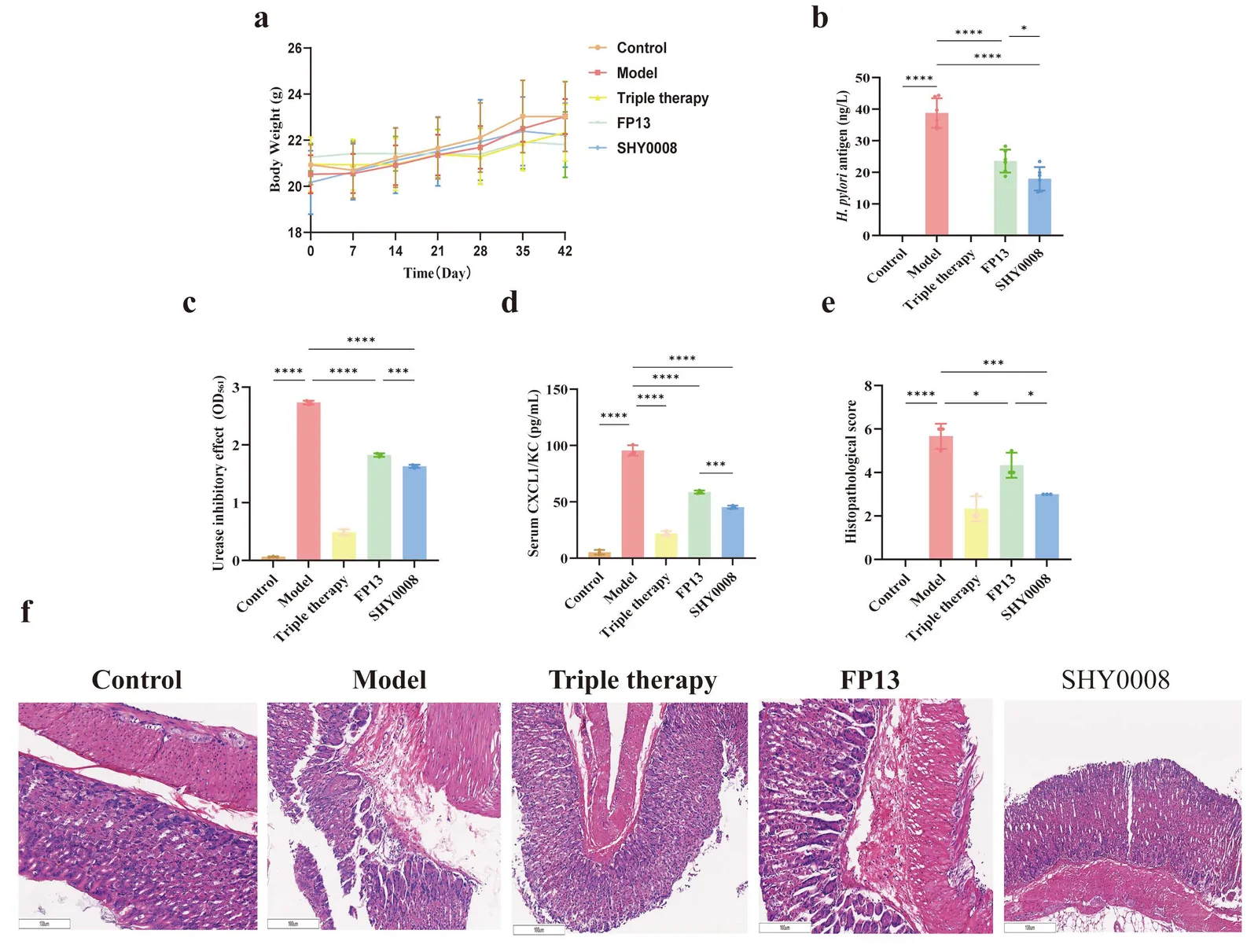
Protective effects of FP13 and SHY0008 against *H. pylori*-associated gastric injury in mice. (**a**) Body-weight changes during the intervention period. (**b**) Gastric *H. pylori* antigen levels. (**c**) Gastric urease activity. (**d**) Serum CXCL1/KC concentrations. (**e**) Histopathological scores of gastric mucosal injury. (**f**) Representative H&E-stained gastric sections. For panel (**a**), n = 6 mice/group and data were analyzed by two-way repeated-measures ANOVA followed by Tukey’s multiple-comparison test. For panel (**b**), n = 6 mice/group; for panels (**c**–**e**), n = 3 mice/group. Panels (**b**–**d**) were analyzed by one-way ANOVA followed by Tukey’s test, and panel (**e**) by the Kruskal–Wallis test followed by Dunn’s test. Data are presented as mean ± SD, except histopathological scores, which are presented as medians with ranges. Statistical significance is indicated as * *p* < 0.05, *** *p* < 0.001 and **** *p* < 0.0001.

All 30 mice included in the formal experiment completed the study, and no animals or data points were excluded from the final analyses. To further evaluate the anti-*H. pylori* activity of FP13 and SHY0008 in a mammalian model, a BALB/c mouse model of *H. pylori* infection was established. During the 4-week intervention period, body weight was monitored weekly. All 30 mice assigned to the formal experiment completed the study, and no animals or data points were excluded from the analyses. Body weight was monitored longitudinally throughout the experimental period. Two-way repeated-measures ANOVA showed a significant effect of time [F(7, 200) = 11.33, *p* < 0.0001], but no significant effect of treatment group [F(4, 200) = 1.388, *p* = 0.2393] and no significant treatment × time interaction [F(28, 200) = 0.6389, *p* = 0.9203]. Tukey’s multiple-comparison test further showed no significant differences in mean body weight among the treatment groups (all adjusted *p* > 0.05; Figure 10a). These findings indicate that body weight changed over time, whereas the overall growth trajectories were comparable among the experimental groups.

Gastric *H. pylori*-associated signals were subsequently evaluated by determining *H. pylori* antigen levels in gastric lavage fluid and urease activity in gastric tissue. As shown in Figure 10b, the gastric *H. pylori* antigen level in the model group was 38.763 ± 4.681, whereas the antigen signal in the blank control group was below the limit of detection. Triple therapy reduced the gastric *H. pylori* antigen signal to below the detection limit. FP13 and SHY0008 treatment also decreased gastric antigen levels to 23.568 ± 3.629 and 17.945 ± 3.690, respectively. Among the probiotic groups, SHY0008 showed a greater reduction in gastric *H. pylori* antigen levels than FP13, whereas triple therapy produced the greatest reduction among all intervention groups.

A similar trend was observed for gastric urease activity (Figure 10c). Urease activity increased markedly from 0.061 ± 0.006 in the blank control group to 2.733 ± 0.033 in the *H. pylori* model group. Triple therapy reduced urease activity to 0.490 ± 0.048, while FP13 and SHY0008 treatment reduced the corresponding values to 1.824 ± 0.030 and 1.629 ± 0.028, respectively. Among the probiotic treatments, SHY0008 showed a greater reduction in gastric urease activity than FP13, although the reduction achieved by triple therapy was more pronounced. Collectively, these findings indicate that SHY0008 reduced two *H. pylori*-associated gastric surrogate markers in infected mice.

The systemic inflammatory response was further assessed by measuring serum CXCL1/KC concentrations. As shown in Figure 10d, serum CXCL1/KC increased from 5.31 ± 1.97 pg/mL in the blank control group to 95.59 ± 4.57 pg/mL in the *H. pylori* model group. Triple therapy markedly reduced serum CXCL1/KC to 21.96 ± 2.06 pg/mL. FP13 and SHY0008 treatment also decreased CXCL1/KC concentrations to 58.65 ± 1.37 and 45.32 ± 1.34 pg/mL, respectively. Triple therapy produced the greatest reduction in circulating CXCL1/KC among the intervention groups, whereas SHY0008 showed a greater reduction than FP13.

Gastric mucosal injury was further evaluated by histopathological scoring and H&E staining. As shown in Figure 10e, the median histopathological score was 0 (range, 0–0) in the blank control group and increased to 6 (range, 5–6) in the *H. pylori* model group. Triple therapy markedly reduced the pathological score to a median of 2 (range, 2–3). FP13 and SHY0008 also improved gastric pathology, with median scores of 4 (range, 4–5) and 3 (range, 3–3), respectively. Among the intervention groups, triple therapy showed the greatest reduction in histopathological injury, whereas SHY0008 exhibited a lower pathological score than FP13.

Representative H&E-stained gastric sections are shown in Figure 10f. The blank control group exhibited intact gastric mucosal architecture, regularly arranged gastric glands, and no obvious inflammatory cell infiltration. In contrast, the *H. pylori* model group showed marked gastric mucosal injury, including disorganized glandular structures, inflammatory cell infiltration, mucosal edema, and vascular congestion. Triple therapy substantially ameliorated these pathological changes. FP13 and SHY0008 treatment also improved gastric mucosal morphology, with reduced inflammatory cell infiltration and better preservation of glandular structure. The improvement observed with SHY0008 was more pronounced than that observed with FP13 and was consistent with the corresponding histopathological scores.

Taken together, these results demonstrate that SHY0008 reduced gastric *H. pylori*-associated antigen and urease signals, attenuated the increase in circulating CXCL1/KC, and ameliorated gastric mucosal injury in infected mice. Although triple therapy produced the strongest overall effect across the evaluated endpoints, SHY0008 consistently showed greater improvement than FP13.

### 3.9. Genome Analysis of Lactiplantibacillus plantarum SHY0008

Whole-genome sequencing of SHY0008 generated 6.32 Gb of raw Illumina paired-end data. After quality filtering, 42,094,498 clean reads comprising 6.24 Gb were retained, corresponding to a clean-data retention rate of 98.86% and an estimated short-read sequencing depth of approximately 1690×. Hybrid assembly produced a draft genome of 3,696,369 bp comprising 656 contigs, with a GC content of 45.24%. The largest contig was 237,812 bp, with an N50 of 56,153 bp and an L50 of 19. Genome annotation predicted 3450 coding sequences, 5 rRNA genes, 71 tRNA genes, and 1 tmRNA.

Genome-based ANI analysis further supported the taxonomic identification of SHY0008 as *Lactiplantibacillus plantarum*. ANI values between SHY0008 and representative *L. plantarum* reference genomes ranged from 98.18% to 98.22%, exceeding the commonly used species-level threshold of approximately 95–96%. This genome-based identification was consistent with the 16S rRNA gene analysis. The SHY0008 genome sequencing project has been deposited in NCBI under BioProject PRJNA1503515 and BioSample SAMN62016464. A GenBank genome assembly accession has not yet been assigned at the time of revision.

**Figure 11:**
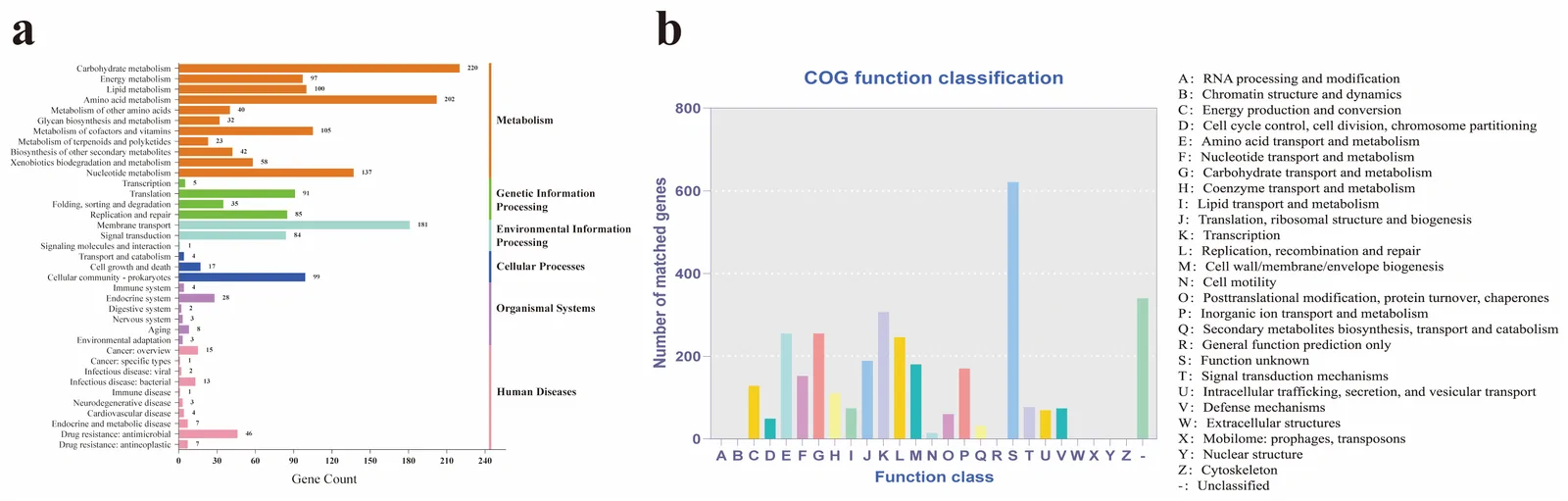
Functional annotation of the SHY0008 genome based on KEGG and COG databases. (**a**) KEGG pathway classification of annotated genes in SHY0008. The six major KEGG functional categories are displayed from top to bottom using different colors, including metabolism, genetic information processing, environmental information processing, cellular processes, organismal systems, and human diseases. (**b**) COG functional classification of SHY0008 genes. Different colors from left to right represent distinct COG functional categories.

The genome of SHY0008 was annotated using the KEGG database. According to KEGG functional classification, the annotated genes were assigned to six major categories, including metabolism, genetic information processing, environmental information processing, human diseases, cellular processes, and organismal systems, containing 1056, 216, 266, 120, 48, and 99 genes, respectively. Within the metabolism category, genes associated with carbohydrate metabolism, amino acid metabolism, and nucleotide metabolism represented the predominant functional groups. In the genetic information processing category, translation-related genes were the most abundant, whereas membrane transport-related genes represented the largest proportion within environmental information processing. These results indicated that SHY0008 possesses robust metabolic activity and efficient material transport capabilities (Figure 11a).

COG annotation further classified 3401 predicted genes into 20 functional categories. The major functional groups included amino acid transport and metabolism (255 genes), carbohydrate transport and metabolism (255 genes), transcription (307 genes), and replication, recombination, and repair (246 genes). These findings suggested that SHY0008 exhibits strong genetic stability and extensive metabolic adaptability (Figure 11b).

Genome annotation identified several plantaricin- or bacteriocin-associated genes involved predominantly in transport, secretion, processing, and membrane-associated functions. These included plnG, plnH, plnV, and plnW, together with an ABC transporter-associated protein, an HlyD family secretion protein, and a CPBP family intramembrane glutamic endopeptidase potentially involved in bacteriocin precursor processing. Targeted examination of canonical plantaricin structural genes showed that plnF was identified (ORF GLOLEKKL_00338), whereas plnA, plnE, plnJ, and plnK were not detected in the current annotation. Therefore, the canonical plnEF and plnJK structural gene pairs were not completely identified, and the present genomic data do not establish the presence of a complete canonical plantaricin biosynthetic locus. Detailed information on these bacteriocin-associated genes and proteins is provided in Table 8.

### 3.10. Genome-Based Safety Assessment

No acquired antimicrobial resistance genes were identified in the SHY0008 draft genome using CARD, ResFinder, or AMRFinderPlus. Likewise, no known virulence-associated genes were detected using VFDB or VirulenceFinder. These findings provide preliminary genome-based evidence that no known acquired resistance or virulence determinants were detected in SHY0008. However, genomic screening alone is insufficient to establish overall probiotic safety. Detailed information is provided in Table S11.

## 4. Discussion

*H. pylori* infection remains a major global health concern, while increasing antibiotic resistance and treatment-associated adverse effects have highlighted the need for effective adjunctive strategies [19]. Probiotics have emerged as promising candidates because of their ability to inhibit pathogen colonization and modulate host–microbe interactions; however, their anti-*H. pylori* effects are highly strain-dependent [13,20,21]. The present study identified *Lactiplantibacillus plantarum* SHY0008 from Miao sour soup through preliminary screening of 12 LAB isolates and evaluated its anti-*H. pylori* potential across complementary in vitro, AGS cell, zebrafish, and murine models. SHY0008 showed the highest mean urease inhibitory activity in the initial screening and subsequently exhibited favorable gastrointestinal tolerance, antibacterial activity, interference with *H. pylori* adhesion, and protective effects against infection-associated tissue injury. Taken together, these findings suggest that SHY0008 represents a promising food-derived probiotic candidate with multiple potential modes of action against *H. pylori*. Importantly, the observed effects should be interpreted as complementary across the different experimental systems rather than as evidence for a single dominant mechanism.

Gastrointestinal tolerance is an important prerequisite for the potential efficacy of probiotic microorganisms. In the present study, SHY0008 maintained viability under simulated gastric conditions in which the artificial gastric juice was initially adjusted to pH 2.0 and the final mixed exposure system had a measured pH of approximately 2.39, indicating favorable tolerance to acidic stress and maintained viability in the presence of 0.3% bile salts, indicating favorable tolerance to bile stresses. Similar tolerance has been reported for other *Lactiplantibacillus plantarum* strains, including NS14, NS16, and NGG [22]. The ability of SHY0008 to withstand these stresses may facilitate its survival during gastrointestinal transit and increase the likelihood of interaction with *H. pylori*. This phenotype may partly reflect adaptation to the acidic and complex microbial environment of Miao sour soup; however, this possibility requires further investigation.

Beyond gastrointestinal tolerance, surface-associated properties may contribute to the probiotic potential of SHY0008. SHY0008 showed greater observed aggregation-related changes and significantly higher cell-surface hydrophobicity than FP13. Because the auto-aggregation and mixed-suspension assays were based on technical replicates from a single bacterial preparation, these findings remain descriptive. Nevertheless, together with the hydrophobicity results, they indicate favorable surface-associated interaction properties [23], although these in vitro parameters should not be interpreted as direct evidence of gastric mucosal colonization.

Urease is a key virulence-associated factor that enables *H. pylori* to survive under acidic gastric conditions by hydrolyzing urea and locally neutralizing gastric acidity. In the present study, SHY0008 inhibited *H. pylori* urease activity by 54.23 ± 3.98%, which was significantly higher than that of the reference strain FP13 (36.62 ± 5.71%). This relatively strong urease inhibitory activity suggests that SHY0008 may interfere with an important acid-adaptation mechanism of *H. pylori*. Similar urease inhibitory effects have also been reported in other probiotic strains, including *Lactiplantibacillus plantarum* Q25 and *Latilactobacillus sakei* LZ217 [24,25]. However, whether urease inhibition directly contributes to the reductions in *H. pylori*-associated gastric signals observed in vivo requires further mechanistic investigation.

SHY0008 produced a 17.56 ± 0.58 mm inhibition zone, comparable to the upper range reported for several probiotic strains against *H. pylori* [25,26], although direct cross-study comparison is limited by differences in diffusion-assay conditions. Activity was also retained in the HTSBP and partially retained after CFS neutralization. Because the HTSBP underwent both heating and sonication, and because pH adjustment does not identify active molecules, these findings do not establish involvement of intact non-viable cells, bacteriocins, or any specific antimicrobial compound. Rather, they suggest that acidity together with additional cell- or supernatant-associated factors may contribute to the observed activity [27,28]. Further compound-level characterization will be required to identify the active components.

In the AGS model, SHY0008 showed greater epithelial adhesion and a larger reduction in the urease-associated *H. pylori* adhesion signal than FP13. Because this colorimetric endpoint is an indirect surrogate rather than a direct viable count, the findings support interference with cell-associated *H. pylori* but require confirmation using direct quantitative methods. In the exploratory IL-8 experiment, SHY0008-treated cells showed a lower observed IL-8 concentration than the *H. pylori* control; however, because only one independent biological experiment was performed, this observation remains descriptive.

The zebrafish model provides a rapid and visually accessible platform for evaluating pathogen-associated signals and host responses, complementing the in vitro and AGS cell models described above. Its optical transparency during early development and suitability for fluorescence-based imaging facilitate dynamic visualization of pathogen-associated signals and tissue responses [29,30]. The zebrafish experiment was designed as a prophylactic pretreatment model rather than a therapeutic model of established infection. FP13, SHY0008, or clarithromycin was administered for 24 h before subsequent exposure to CM-DiI-labeled *H. pylori*. Under this design, SHY0008 pretreatment was associated with lower intestinal *H. pylori*-associated fluorescence and reduced histopathological injury compared with the untreated exposure group. Clarithromycin produced the lowest fluorescence intensity among the intervention groups, whereas SHY0008 showed a lower observed fluorescence signal than FP13. Because the quantified larvae within each group were sampled from a single treatment well, these findings were interpreted descriptively. Therefore, the zebrafish results support a potential prophylactic protective effect of SHY0008 against subsequent *H. pylori* exposure, but they do not demonstrate therapeutic efficacy against an established *H. pylori* infection.

In the BALB/c model, SHY0008 reduced gastric *H. pylori*-associated antigen and urease signals, serum CXCL1/KC, and gastric mucosal injury [31,32,33,34,35]. These effects were generally greater than those observed with FP13, whereas triple therapy produced the strongest overall improvement, supporting SHY0008 as a complementary rather than replacement strategy for conventional eradication therapy. *H. pylori* ATCC 43504 (NCTC 11637) is a human-derived rather than conventional mouse-adapted strain, and the gastric antigen and urease endpoints are indirect surrogate markers rather than direct measures of viable bacterial burden [36]. Direct assessment of viable gastric persistence will require selective culture and/or validated quantitative molecular approaches.

Whole-genome analysis provided further genomic insights into the probiotic and antimicrobial potential of SHY0008. KEGG and COG annotation revealed a broad repertoire of genes involved in carbohydrate and amino acid metabolism, membrane transport, transcription, and DNA replication and repair, indicating substantial metabolic versatility and environmental adaptability. Notably, genome mining identified a plantaricin-associated gene module comprising plnG, plnH, plnV, plnW, ABC transporter-associated genes, hlyD, and a CPBP family protein. The identified genes indicate the presence of bacteriocin-associated transport, processing, secretion, and protection functions. However, among the canonical plantaricin structural genes examined, only plnF was detected, whereas plnA, plnE, plnJ, and plnK were not identified. Therefore, the genomic data do not support the presence of a complete canonical plnEF/plnJK plantaricin system, and they do not by themselves demonstrate plantaricin production. Further transcriptomic, proteomic, and compound-level analyses are required to determine whether bacteriocin-like antimicrobial peptides contribute to the anti-*H. pylori* activity of SHY0008.

Genome-based ANI analysis further supported assignment of SHY0008 to *L. plantarum*, with ANI values of 98.18–98.22% against representative genomes. Preliminary genome-based safety screening detected no known acquired antimicrobial-resistance genes or virulence-associated determinants. However, genomic screening alone cannot establish comprehensive probiotic safety, and phenotypic antimicrobial susceptibility and additional functional safety assessments remain necessary.

## 5. Limitations

Several limitations should be considered. First, the aggregation assays were based on technical replicates from a single bacterial preparation, and the AGS IL-8 experiment included only one independent biological experiment; these findings therefore remain descriptive. The zebrafish experiment similarly used a single treatment well per group, with individual larvae serving as within-well subsamples. Second, the CFS assay did not include a pH-matched medium control or longitudinal monitoring of mixture pH, limiting separation of acidity from other CFS-associated effects. Third, the murine study used the human-derived *H. pylori* ATCC 43504 strain, was not based on a formal a priori power calculation, and relied on antigen and urease measurements as indirect infection-associated markers rather than direct viable bacterial quantification. Finally, genome-based safety screening and the fragmented draft assembly cannot establish comprehensive probiotic safety or confirm production of the predicted plantaricin-associated products. Independent replication, direct bacterial quantification, phenotypic safety testing, and compound-level characterization are warranted.

## 6. Conclusions

This study identified Miao sour soup-derived *Lactiplantibacillus plantarum* SHY0008 as a probiotic candidate with anti-*H. pylori* and gastroprotective potential. Across complementary in vitro, cellular, zebrafish, and murine models, SHY0008 showed gastrointestinal tolerance, antagonistic activity against *H. pylori*-associated endpoints, reduced epithelial adhesion signals and inflammatory responses, and alleviated tissue injury. Genome analysis supported its taxonomic identification and provided preliminary functional and safety information, but did not establish production of specific antimicrobial compounds or comprehensive probiotic safety. These findings support further investigation of SHY0008 as a food-derived adjunctive probiotic candidate for *H. pylori* management.

## Figures and Tables

**Table 1:** Experimental design of the prophylactic zebrafish *H. pylori* exposure model.

Group	Number of Larvae	Treatment
Blank control	30	Embryo medium only
*H. pylori* model	30	5 × 10^7^ CFU/mL *H. pylori*
Clarithromycin	30	5 × 10^7^ CFU/mL *H. pylori* + 125 μg/mL Clarithromycin
FP13	30	5 × 10^7^ CFU/mL *H. pylori* + 1 × 10^8^ CFU/mL FP13
SHY0008	30	5 × 10^7^ CFU/mL *H. pylori* + 1 × 10^8^ CFU/mL SHY0008

**Table 2:** Experimental design of the probiotic intervention in the *H. pylori*-infected mice model.

Group	Number of Mice (n)	Treatment
Blank control	6	Non-infected mice received sterile normal saline by oral gavage (0.5 mL/mouse/day) for 4 weeks.
*H. pylori* model	6	*H. pylori*-infected mice received sterile normal saline by oral gavage (0.5 mL/mouse/day) for 4 weeks.
Positive control	6	*H. pylori*-infected mice received the positive drug by oral gavage (0.5 mL/mouse/day) for 2 weeks, followed by sterile normal saline (0.5 mL/mouse/day) for an additional 2 weeks.
FP13	6	*H. pylori*-infected mice received FP13 (1 × 10^9^ CFU/mL) by oral gavage (0.5 mL/mouse/day) for 4 weeks.
SHY0008	6	*H. pylori*-infected mice received SHY0008 (1 × 10^9^ CFU/mL) by oral gavage (0.5 mL/mouse/day) for 4 weeks.

**Table 3:** Molecular and genome-based taxonomic identification of SHY0008.

Subject Strain	Reference Taxon	Reference Strain/Status	Genome Accession	ANI (%)
SHY0008	*Lactiplantibacillus* *plantarum*	*L. plantarum* subsp. *plantarum* ATCC 14917, type strain	GCA_000143745.1	98.22
SHY0008	*Lactiplantibacillus* *plantarum*	Reference genome	GCA_014131735.1	98.22
SHY0008	*Lactiplantibacillus* *plantarum*	*L. plantarum* subsp. plantarum ATCC 14917	GCA_001434175.1	98.19
SHY0008	*Lactiplantibacillus* *plantarum*	Reference genome	GCA_000615325.1	98.18

**Table 4:** Inhibitory effects of VBS and HTSBP of FP13 and SHY0008 against *H. pylori* growth.

Strain	Inhibition Zone Diameter of VBS (mm)	Inhibition Zone Diameter of HTSBP (mm)
Control	8.00 ± 0.00	8.00 ± 0.00
FP13	15.39 ± 0.29	14.79 ± 0.81
SHY0008	17.56 ± 0.58	17.21 ± 0.29

**Table 5:** Inhibitory effects of CFSs from FP13 and SHY0008 on *H. pylori* growth.

Strain	OD_600_-Based Reduction Rate of NCFS Against *H. pylori* (%)	OD_600_-Based Reduction Rate of PACFS Against *H. pylori* (%)
FP13	34.31 ± 1.81	28.80 ± 1.60
SHY0008	43.98 ± 4.50	38.07 ± 1.76

**Table 8:** Identification of plantaricin-associated biosynthetic and secretion-related proteins in SHY0008.

Gene/Protein	Protein Length (aa)	Best Hit Organism	Identity (%)	Query Coverage (%)	E-Value	Accession
*plnG*	323	*Lactiplantibacillus* *plantarum*	98.96%	100%	2 × 10^−57^	MCG0626676.1
ABC transporter associated protein	716	*Lactiplantibacillus* *plantarum*	99.01%	100%	0.0	WP_262879939.1
HlyD family secretion protein	464	*Lactiplantibacillus* *plantarum*	98.06%	100%	0.0	WP_063722042.1
*plnH*	398	*Lactiplantibacillus* *plantarum*	98.49%	87%	0.0	WP_348001222.1
CPBP family intramembrane glutamic endopeptidase	228	*Lactiplantibacillus* *plantarum*	99.56%	100%	9 × 10^−161^	WP_222893288.1
*plnV*	185	*Lactiplantibacillus* *plantarum*	98.38%	82%	4 × 10^−127^	MCG0632750.1
*plnW*	142	*Lactiplantibacillus* *plantarum*	95.07%	62%	6 × 10^−89^	MCG0632751.1

## Data Availability

The SHY0008 genome sequencing project has been registered in NCBI under BioProject PRJNA1503515 and BioSample SAMN62016464. A GenBank genome assembly accession had not yet been assigned at the time of revision.

## Supplementary Materials

The following supporting information can be downloaded at https://www.mdpi.com/article/10.3390/microorganisms14102387/s1, Table S1. Formulation of MRS Broth Medium for the Cultivation of Lactic Acid Bacteria; Table S2. Formulation of MRS Agar Medium for the Cultivation of Lactic Acid Bacteria; Table S3. Formulation of *Helicobacter pylori* Broth (Liquid Medium); Table S4. Formulation of *Helicobacter pylori* Medium (Agar); Table S5. Formulation of AGS Cell Culture Media; Table S6. Phenotypic and taxonomic characteristics of LAB isolates recovered from Miao sour soup; Table S7. Preliminary screening of LAB isolates based on inhibition of *H. pylori* urease activity; Table S8. Composition and preparation of urease indicator solution; Table S9. Enterocolitis score system for histology evaluation; Table S10. Semi-quantitative Histopathological Scoring Criteria for Gastric Mucosal Inflammation (Modified Updated Sydney System); Table S11. Genome-based safety assessment of *Lactiplantibacillus plantarum* SHY0008; Table S12. Abbreviations. File S1. Preparation procedure for simulated gastric fluid used in the acid-tolerance assay.
