# Supplementary material for: Anti-Helicobacter pylori Activity and Gastroprotective Effects of Miao Sour Soup-Derived Lactiplantibacillus plantarum SHY0008
Source: Microorganisms. 2026 Oct 19;14(10):2387. doi: 10.3390/microorganisms14102387 (PMC13707997; doi:10.3390/microorganisms14102387)
Supplement: Supplementary file 1 [file microorganisms-14-02387-s001.zip › microorganisms-4530578-supplementary.pdf]

File S1. Preparation procedure for simulated gastric fluid used in the acid-tolerance assay.

Preparation of simulated gastric fluid: Eighty milliliters of deionized water was measured, and 0.2 g of sodium chloride was added and stirred until completely dissolved. Concentrated hydrochloric acid was then added dropwise with continuous stirring, and the pH was adjusted to 2.0 using a calibrated electronic pH meter. Subsequently, 0.32 g of pepsin was added and dissolved completely. The final volume was adjusted to 100 mL with deionized water and mixed thoroughly. The solution was filtered through a 0.22 μm membrane filter to remove undissolved particulates, stored at 4 °C, and used within 24 h.

Table S1. Formulation of MRS Broth Medium for the Cultivation of Lactic Acid Bacteria

Component	Concentration（g/L）
Glucose	20
Casein peptone	10
Beef extract	10
Sodium acetate	5
Yeast extract	4
Tri-ammonium citrate	2
K_2HPO_4	2
Tween 80	1
$MgSO_4 \cdot 7H_2O$	0.2
$MnSO_4 \cdot 4H_2O$	0.05

Note: The medium was adjusted to pH 5.7 ± 0.2 before sterilization (121 °C, 15 min)

Table S2. Formulation of MRS Agar Medium for the Cultivation of Lactic Acid Bacteria

Component	Concentration（g/L）
Glucose	20
Agar	15
Casein peptone	10
Beef extract	10
Sodium acetate	5
Yeast extract	4
Tri-ammonium citrate	2
K_2HPO_4	2
Tween 80	1
$MgSO_4 \cdot 7H_2O$	0.2
$MnSO_4 \cdot 4H_2O$	0.05

Note: The medium was adjusted to pH 6.2 ± 0.2 before sterilization (121 °C, 15 min)

Table S3. Formulation of *Helicobacter pylori* Broth (Liquid Medium)

Component	Concentration（g/L）
Proteose peptone	10.0
Bovine brain infusion powder	10.0
Bovine heart infusion powder	9.0
Sodium chloride	5.0
Disodium hydrogen phosphate	2.5
D-glucose (Dextrose)	2.0

Note: The medium was adjusted to pH 7.4 ± 0.2 before sterilization (121 °C, 15 min)，Aseptically add 1 vial of selective supplement and 7 mL of defibrinated sheep blood per 93 mL of basal medium.The *H. pylori* selective supplement contains 1 mg of nalidixic acid, 0.5 mg of trimethoprim (TMP), 0.3 mg of vancomycin and 0.2 mg of amphotericin B per vial, which is used to inhibit contaminating bacteria and fungi in the culture system.

Table S4. Formulation of *Helicobacter pylori* Medium (Agar)

Component	Concentration（g/L）
Proteose peptone	10.0
Bovine brain infusion powder	10.0
Bovine heart infusion powder	9.0
Sodium chloride	5.0
Disodium hydrogen phosphate	2.5
D-glucose (Dextrose)	2.0
Agar	15.0

Note: The medium was adjusted to pH 7.4 ± 0.2 before sterilization (121 °C, 15 min)，Aseptically add 1 vial of selective supplement and 7 mL of defibrinated sheep blood per 93 mL of basal medium. The *H. pylori* selective supplement contains 1 mg of nalidixic acid, 0.5 mg of trimethoprim (TMP), 0.3 mg of vancomycin and 0.2 mg of amphotericin B per vial, which is used to inhibit contaminating bacteria and fungi in the culture system.

Table S5. Formulation of AGS Cell Culture Media

Medium Type	Components	Volume Concentration (v/v)	Storage & Application
Complete AGS Cell Medium	Ham's F-12K Nutrient Mixture<br>Fetal Bovine Serum(FBS)<br>Penicillin-Streptomycin -Amphotericin B Solution (PSA)	89%<br>10%<br>1%	Stored at 4 °C after thorough mixing; for routine cell maintenance and subculture
Experimental AGS Cell Medium	Ham's F-12K Nutrient Mixture<br>Fetal Bovine Serum (FBS)	90%<br>10%	Stored at 4 °C after thorough mixing; for H. pylori co-culture assays (antibiotic-free to avoid interference with bacterial activity)

Table S6. Phenotypic and taxonomic characteristics of LAB isolates recovered from Miao sour soup.

**Strain No.**	**Strain Species and Genus**	**Cell Morphology**	**Staining Result**	**Colony Morphology**
SHY0001	*Lacticaseibacillus paracasei*	Short rod-shaped	G+	Milky white, small-sized, round and convex, smooth surface, regular margin
SHY0002	*Lentilactobacillus parabuchneri*	Short rod-shaped	G+	Milky white, small-sized, round and convex, smooth surface, regular margin
SHY0003	*Lactobacillus harbinensis*	Long rod-shaped	G+	Milky white, small-sized, round and convex, smooth surface, regular margin
SHY0004	*Limosilactobacillus fermentum*	Short rod-shaped	G+	Grayish white, large-sized, round and convex, moist surface, irregular margin
SHY0005	*Lactobacillus harbinensis*	Short rod-shaped	G+	Grayish white, small-sized, round and convex, smooth surface, regular margin
SHY0006	*Limosilactobacillus fermentum*	Long rod-shaped	G+	Milky white, large-sized, round and convex, moist surface, irregular margin
SHY0007	*Limosilactobacillus fermentum*	Short rod-shaped	G+	Milky white, small-sized, round and convex, smooth surface, regular margin
SHY0008	*Lactiplantibacillus plantarum*	Short rod-shaped	G+	Milky white, small-sized, round and convex, smooth surface, regular margin
SHY0009	*Lacticaseibacillus paracasei*	Long rod-shaped	G+	Milky white, small-sized, round and convex, smooth surface, regular margin
SHY00010	*Limosilactobacillus fermentum*	Short rod-shaped	G+	Grayish white, large-sized, round and convex, moist surface, irregular margin
SHY00011	*Lacticaseibacillus paracasei*	Short rod-shaped	G+	Grayish white, large-sized, round and convex, moist surface, irregular margin
SHY00012	*Limosilactobacillus fermentum*	Short rod-shaped	G+	Grayish white, large-sized, round and convex, moist surface, irregular margin

Table S7. Preliminary screening of LAB isolates based on inhibition of *H. pylori* urease activity.

Strain No.	Mean OD_{561}
SHY0001	1.410 ± 0.187
SHY0002	1.479 ± 0.064
SHY0003	0.827 ± 0.073
SHY0004	1.673 ± 0.119
SHY0005	1.257 ± 0.039
SHY0006	1.247 ± 0.057
SHY0007	1.356 ± 0.057
SHY0008	0.699 ± 0.059
SHY0009	0.900 ± 0.056
SHY00010	1.035 ± 0.037
SHY00011	1.033 ± 0.059
SHY00012	1.229 ± 0.047

Table S8. Composition and preparation of urease indicator solution

Component	Final concentration	Amount for 100 mL preparation	Preparation procedure
Sodium chloride (NaCl)	0.9% (w/v)	0.9 g	Dissolved in sterile distilled water to prepare 0.9% NaCl solution
Urea	20 mmol/L	120 mg	Dissolved in sterile 0.9% NaCl solution
Phenol red	14 μg/mL	1.4 mg	Added and dissolved in the above solution as a pH indicator
Hydrochloric acid (HCl)	1.0 M	—	Used to adjust the pH of the solution to 6.5
Sterilization	—	—	The prepared solution was sterilized by filtration through a 0.22 μm sterile membrane filter and used immediately

Table S9. Enterocolitis score system for histology evaluation

Numerical score	Impaired epithelial integrity	Expanded clefts/reduced projections	Expanded gut lumen	Goblet cell appearance
0	Normal	Normal	Normal	Normal
1	Slight disruption	Slight disruption	Slight expansion	Increased in number
2	Moderate disruption	Moderate disruption	Moderate expansion	Increased in number and different in sizes
3	Severe disruption	Severe disruption	Severe expansion	Severe morphological changes

Table S10. Semi-quantitative Histopathological Scoring Criteria for Gastric Mucosal Inflammation (Modified Updated Sydney System)

Scoring Indicator	0 (Normal)	1 (Mild)	2 (Moderate)	3 (Severe)
Inflammatory cell infiltration	No obvious inflammatory cell infiltration in the mucosal layer; normal tissue architecture	Scattered inflammatory cells in the superficial mucosa, involving less than 1/3 of the mucosal layer	Multifocal inflammatory cell infiltration in the mucosa and submucosa, involving 1/3 to 2/3 of the mucosal layer	Diffuse severe inflammatory cell infiltration across the full mucosal layer, involving more than 2/3 of the mucosa and potentially extending to the serosa
Epithelial injury	Intact and continuous epithelial layer with regular cell morphology; no exfoliation or defects	Mild degeneration of superficial epithelium with focal minor epithelial exfoliation	Epithelial cell degeneration and necrosis; focal exfoliation and defects involving the full epithelial layer	Extensive epithelial necrosis and exfoliation; mucosal erosion or ulcer formation
Gland destruction	Neatly arranged glands with uniform size and intact structure	Mildly disorganized gland arrangement; no obvious gland atrophy or reduction in number	Disordered gland structure; focal atrophy and reduced gland number with irregular morphology	Severe gland destruction with widespread atrophy; loss of gland architecture, with visible intestinal metaplasia or cystic dilation
Mucosal edema	No edema; normal interstitial space	Mild mucosal edema with slightly widened interstitial space	Moderate edema in the mucosa and submucosa with significantly widened interstitial space	Severe full-thickness edema; loose and separated tissue with markedly widened interstitial space

Table S11. Genome-based safety assessment of *Lactiplantibacillus plantarum* SHY0008

Database	Target	Result	Interpretation
CARD	Acquired antimicrobial resistance genes	No hit	No acquired antimicrobial resistance genes detected
ResFinder	Antimicrobial resistance genes	No hit	No acquired antimicrobial resistance genes detected
AMRFinderPlus	Antimicrobial resistance genes	No hit	No acquired antimicrobial resistance genes detected
VFDB	Virulence factors	No hit	No known virulence factors detected
VirulenceFinder	Virulence genes	No hit	No virulence-associated genes detected

Table S12. Abbreviations

Abbreviation	English Full Name
H. pylori	*Helicobacter pylori*
SHY0008	*Lactiplantibacillus plantarum* SHY0008
IL-8	interleukin-8
WHO	World Health Organization
CagA	Cytotoxin-associated gene A
VacA	Vacuolating cytotoxin A
NF-κB	nuclear factor-κB
FP13	*Streptococcus thermophilus* FP13
CFS	Cell-free supernatants
MOI	Multiplicity of infection
